# Identification of hexosamine biosynthesis pathway as a novel prognostic signature and its correlation with immune infiltration in bladder cancer

**DOI:** 10.3389/fmolb.2022.1009168

**Published:** 2022-09-08

**Authors:** Yangyan Cui, Hanyi Feng, Jiakuan Liu, Jiajun Wu, Rujian Zhu, Ruimin Huang, Jun Yan

**Affiliations:** ^1^ Model Animal Research Center, Nanjing University, Nanjing, China; ^2^ School of Chinese Materia Medica, Nanjing University of Chinese Medicine, Nanjing, China; ^3^ Shanghai Institute of Materia Medica, Chinese Academy of Sciences, Shanghai, China; ^4^ Department of Laboratory Animal Science, Fudan University, Shanghai, China; ^5^ Department of Urology, Shanghai Pudong Hospital, Fudan University Pudong Medical Center, Shanghai, China; ^6^ MOE Key Laboratory of Model Animals for Disease Study, Model Animal Research Center of Nanjing University, Nanjing, China

**Keywords:** hexosamine biosynthesis pathway, bladder cancer, prognosis, immune infiltration, risk score

## Abstract

**Background:** Urinary bladder cancer (UBC) is one of the common urological malignancies, lacking reliable biomarkers to predict clinical outcomes in UBC patients. Thus, it is needed to identify the novel diagnostic/prognostic biomarkers to stratify the high-risk UBC patients. As a shunt pathway of glycolysis, the hexosamine biosynthesis pathway (HBP) has been implicated in carcinogenesis. However, its prognostic value in UBC remains unclear.

**Methods:** The RNA sequencing and mRNA microarray datasets were downloaded from The Cancer Genome Atlas (TCGA) and the Gene Expression Omnibus databases. The expression levels of five HBP genes were analyzed in normal and UBC samples, and their associations with stage, grade and survival were plotted. The performance of HBP risk group was evaluated by receiver-operating characteristics (ROC) curve. The HBP signature was generated by Gene Set Variation Analysis (GSVA) and its association with clinicopathological parameters and survival were analyzed. Gene Ontology (GO) and Kyoto Encyclopedia of Genes and Genomes (KEGG) analyses were carried out to examine the potential biological functions of HBP using DAVID online tool. The infiltration estimation fraction of immune cells was performed using CIBERSORT-ABS algorithm. Gene set enrichment analysis (GSEA) was used to explore the potential function of HBP in tumor immunoregulation.

**Results:** Four HBP genes were upregulated in UBCs compared to normal tissues in TCGA-BLCA dataset. The upregulation of all five HBP genes was significantly associated with tumor grade and stage of UBC in three independent UBC datasets. The expression of HBP genes predicted poor clinical outcomes in UBC patients in both TCGA-BLCA and GSE13507 datasets. The high-risk group based on HBP genes showed a poor prognosis. Furthermore, HBP signature was positively associated with tumor grade and stage in TCGA-BLCA dataset and with tumor grade, stage, distal metastasis and poor survival in GSE13507 dataset. Interestingly, high-HBP signature group exhibited a high infiltration of immune cells, particularly the macrophage population.

**Conclusion:** We identified that HBP was a promising prognostic biomarker in UBC patients and strongly associated with immune infiltration.

## Introduction

Urinary bladder cancer (UBC) is a malignant tumor that seriously endangers health in the human urinary system. In 2022, 81,180 newly diagnosed UBC cases in the United States are estimated, with 17,100 deaths (Siegel et al., 2022). Approximately 75% of UBC patients are diagnosed with non-muscle-invasive bladder cancer (NMIBC), and the other 25% are muscle-invasive bladder cancer (MIBC) with poorer clinical outcomes than NMIBC (Sanli et al., 2017). Unfortunately, 20% of NMIBC patients will eventually develop into MIBC. Therefore, it is urgent to identify the UBC patients with high risk of poor prognosis at molecular level, in addition to histology analysis.

The metabolism of cancer cells is different from that of normal corresponding cells, partially due to the genetic alterations of oncogene and tumor suppressor genes (Kerk et al., 2021; Pavlova et al., 2022). The aerobic glycolysis is regarded as one of the cancer metabolic hallmarks, which is also called as Warburg effect to build up biomass in the need of highly proliferative cancer cells (Martínez-Reyes and Chandel, 2021). The tumor suppressor p53 inhibits glycolysis by reducing the expression of glucose transporter GLUT1 and downstream glycolytic enzymes, such as hexokinases, glucose-6-phosphate isomerase, etc, while the activations of c-Myc and hypoxia-inducible factor 1α (HIF1α) can induce the transcriptions of these glycolysis-related genes (Boutelle and Attardi, 2021; Elzakra and Kim. 2021; Tambay, et al., 2021). In UBC development, we previously revealed that the overexpression of SRC-3/AIB1 oncoprotein functions as a coactivator for HIF1α to promote glycolysis in UBC cells (Zhao, et al., 2014). In addition, loss of the tumor suppressor p15^INK4B^ released its inhibitory effects on the glycolytic protein Enolase 1 and eventually promoted glycolysis. As a result, p15^INK4B^ deficiency in mouse urothelial cells induced an early-onset of UBC development in the presence of oncogenic HRAS (Xia, et al., 2021). Altogether, cumulative evidence indicates that elevated aerobic glycolysis is a common phenomenon in UBC cells.

Though the majority of glucose enters the glycolytic pathway, roughly 2%–5% of glucose will be shunted to the hexosamine biosynthesis pathway (HBP) (Akella et al., 2019). Incorporated with a small fraction of glutamine, acetyl-CoA and UTP through four consecutive enzymatic steps, fructose-6-phosphate, the intermediate metabolite in glucose metabolism, eventually converts into uridine diphosphate N-acetylglucosamine (UDP-GlcNAc) as the end product of HBP. Since UDP-GlcNAc serves as a donor substrate for protein O-GlcNAcylation, as well as O- and N-linked glycosylation, HBP functions as a sensor for nutrients, linking cell metabolism and cellular signaling (Metallo and Vander Heiden, 2010; Vasseur and Manié, 2015; Chiaradonna et al., 2018; Parker et al., 2021). Meanwhile, in UBC samples various glycan changes have been frequently detected, which include truncation/increased branching of O-glycans, fucosylation, and sialylation (Jian et al., 2020). Consistently, our previous study revealed that a unique glycogene cluster, covering the glycosyltransferases with O-link and N-link glycosylation, could distinguish the UBC patients with poor prognosis from the whole cohort (Dalangood et al., 2021). Among the list of the gene signature, the overexpression of ST3GAL6 was related to the levels of α2,6-sialylation in basal subtype of UBC cells. Moreover, targeting the β-galactoside-binding protein (galectin-3) by modified citrus pectin could induce UBC cell apoptosis and inhibit tumorigenicity (Fang et al., 2018). Overall, the alterations of different glycans during bladder carcinogenesis suggest that the availability of these sugar donors may also reflect, and even determine, the statuses of cancer cells in response to various intracellular alterations and extracellular cues. Therefore, we hypothesized the pathways for sugar donor production might be important to tumorigenesis and progression.

There exist five enzymes in HBP, including glutamine:fructose-6-phosphate amidotransferase 1 and 2 (GFPT1 and 2, also known as GFAT1 and 2), glucosamine-6-phosphate N-acetyltransferase 1 (GNPNAT1), phosphoglucomutase 3 (PGM3) and UDP-N-acetylglucosamine pyrophosphorylase 1 (UAP1; Lam et al., 2021). The dysregulation of some HBP genes in cancer development is quite cancer-type dependent. For example, GFPT1 was upregulated in prostate, colon and pancreatic cancers, but downregulated in gastric cancer (Itkonen et al., 2013; Duan et al., 2016; Yang et al., 2016; Vasconcelos-Dos-Santos et al., 2017). Hence, it is necessary to comprehensively investigate whether HBP is activated during UBC development and whether its activation can be served as a prognostic indicator.

In this study, we carried out bioinformatics analysis to examine the five HBP genes in four independent databases, including TCGA-BLCA, GSE13507, GSE3167, and GSE32894 datasets. The potential of cancer risk of HBP and the association of HBP with inflammation were also investigated.

## Materials and methods

### Data collection

To explore the association between HBP genes and UBC, the Cancer Genome Atlas-Bladder Urothelial Carcinoma (TCGA-BLCA) dataset (involving 19 normal tissues and 406 UBC tissues) was downloaded from the Cancer Genome Atlas (https://portal.gdc.cancer.gov/). We also downloaded another three independent datasets, ie., GSE13507 (69 normal tissues and 188 UBC tissues; Kim et al., 2010), GSE3167 (14 normal tissues and 46 UBC tissues; Dyrskjøt et al., 2004) and GSE32894 (308 UBC tissues; Sjödahl et al., 2012) from the Gene Expression Omnibus (GEO) database (https://www.ncbi.nlm.nih.gov/geo/). Clinical data of UBC samples in TCGA dataset (TCGA-BLCA) were downloaded from Genomic Data Commons Data Portal (http://www.cbioportal.org/index.do).

### Survival analysis

For survival analysis, the samples were divided into low and high groups based on HBP genes expression levels or risk score levels. The differences in overall survival (OS), disease-free survival (DFS) and cancer-specific survival (CSS) between the high-risk and low-risk groups were estimated using the Kaplan-Meier method. Survival probability was calculated using Log-rank test. The significance threshold was defined as *p* < 0.05.

### Construction and evaluation of the prognosis model

With rms package for R software, the nomograms were established by incorporating *GFPT1*, *GFPT2*, *GNPNAT1*, *PGM3*, and *UAP1* mRNA expression characteristics, and concordance index (C-index) was used to measure the predictive accuracy of the nomogram. Using the bootstrap method (1,000 replicates), we created calibration curves to visualize the deviation of predicted probabilities from the actual values. COX model was used to integrate the prognostic value of the five genes into a five-gene signature model for UBC. The risk score of each sample was calculated as follows: risk score = β1x1 + β2x2 + … + βixi. In this formula, xi and βi indicate the mRNA expression level and the risk coefficient of each gene, respectively. Then, UBC cohorts were divided into high- and low-risk groups based on the median risk score. The receiver-operating characteristic (ROC) curves were created *via* timeROC package to evaluate the prognostic accuracy of the risk score.

### The correlation analysis

To examine the association among HBP signature scores, five HBP genes and clinicopathological variables, HBP signature score of each sample in TCGA-BLCA and another two GEO datasets were analyzed using Gene Set Variation Analysis (GSVA). In brief, the HBP signature scores of samples in these datasets were obtained by the “GSVA” R package, using *GFPT1*, *GFPT2*, *GNPNAT1*, *PGM3*, and *UAP1* as HBP markers. The samples were divided by the median GSVA value and then plotted as a heatmap between GSVA value and other information in Morpheus (https://software.broadinstitute.org/morpheus). The Chi-square test was used to determine if they were correlated.

The correlations between HBP signature/five HBP members (*GFPT1*, *GFPT2*, *GNTNAP1*, *PGM3*, and *UAP1*), and infiltrating estimation fraction of immune cells/M2-macrophage associated genes were evaluated by the “corrplot” R package. These correlations were presented by heatmap, using Cluster 3.0 software, or visualized using “corrplot” R package.

### Estimation of immune cell infiltration

The immune cell infiltrating estimation fraction for TCGA-BLAC by CIBERSORT-ABS (including 22 immune cell types) algorithm was downloaded from TIME2.0 (http://timer.cistrome.org). The “corrplot” R package was used to evaluate the Pearson correlation between infiltrating immune cells and HBP signature or the expression of five HBP members. These correlations were presented by heatmap, using Cluster 3.0 software.

### Functional enrichment analysis

1,293 co-expressed genes with HBP in TCGA-BLCA dataset were ascertained by Pearson correlation analysis using r ≥ 0.25 as the cutoff value (Supplementary Table S1). The DAVID online tool (https://www.david.ncifcrf.gov/) was used to perform the Gene Ontology (GO) and Kyoto Encyclopedia of Genes and Genomes (KEGG) enrichment analyses for the co-expressed genes with HBP in TCGA-BLCA dataset. The *p* value < 0.05 served as the cutoff value, and these GO terms or KEGG pathways were considered to be significantly enriched. The top GO terms and KEGG pathways were presented by bubble diagrams.

### Gene set enrichment analysis

The C7 Immunologic signature gene sets V7.5 contain 5,219 gene sets for GSEA (software version 4.2.3) evaluation of the relationship between HBP and immunologic signature. The top 10 signature positively associated with HBP was shown in Supplementary Table S2. The nominal *p* < 0.05 and FDR q < 0.25 were use as cutoff values.

### Statistical analyses

Student’s *t*-test was used to detect whether there was a difference in tissue type (normal and cancer), tumor stage (low and high), and tumor grade (low and high) of TCGA-BLCA, GSE3167, GSE13507, and GSE32894 datasets, respectively. Nomograms were constructed based on the independent factors of COX multivariate analyses in GSE13507 and GSE32894 datasets. The concordance index (C-index) and calibration were also assessed to effectively measure the performance of constructed nomograms. The samples were divided by the median GSVA value, using *GFPT1*, *GFPT2*, *GNPNAT1*, *PGM3*, and *UAP1* as HBP markers. Correlation analysis between variables was conducted by Pearson correlation analysis. Student’s *t*-test was used to compare data between two groups. The Chi-square test was used to determine the correlations between GSVA value and other clinicopathological parameters. The significant differences in different clinic information between the high and low GSVA value groups were analyzed using GraphPad Prism 9. Statistical significance was defined as *p* < 0.05. (*, *p* < 0.05, **, *p* < 0.01, ***, *p* < 0.001, and ns, non-significant).

## Results

### The genetic alterations and upregulation of hexosamine biosynthesis pathway genes in urinary bladder cancer

**FIGURE 1:**
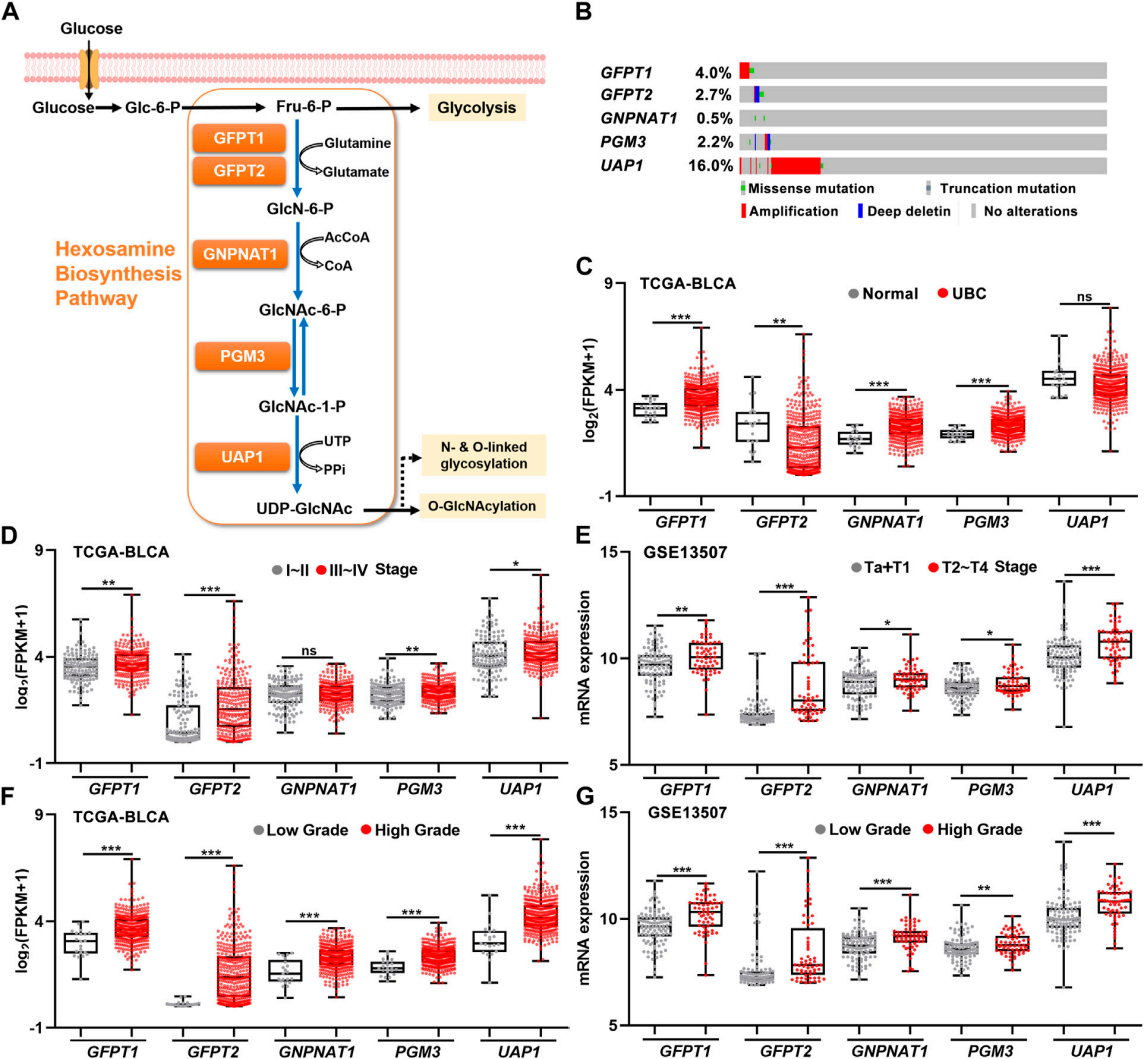
The relationships between gene expression levels of five HBP genes and clinicopathological factors in UBC patients. **(A)** Schematic illustration of the HBP with five key HBP enzymes, including *GFPT1*, *GFPT2*, *GNPNAT1*, *PGM3,* and *UAP1*. **(B)** The genetic alterations of five HBP genes in human UBC patients derived from TCGA dataset. **(C)** The mRNA levels of five HBP genes in normal bladder tissues (*n* = 19) and UBC tissues (*n* = 406) in TCGA-BLCA dataset. **(D,E)** The mRNA levels of five HBP genes in low stage (stage I–II, *n* = 131; or Ta + T1, *n* = 104) and high stage (stage III–IV, *n* = 273; or T2–T4, *n* = 61) of UBCs from TCGA-BLCA **(D)** and GSE13507 **(E)**, respectively. **(F,G)** The mRNA levels of five HBP genes in low- and high-grade of UBCs from TCGA-BLCA **(F)**; Low Grade (*n* = 21) vs*.* High Grade (*n* = 382), and GSE13507 **(G)**; Low Grade (*n* = 105) vs*.* High Grade (*n* = 60), respectively. *p < 0.05, **p < 0.01, ***p < 0.001, and ns, non-significant.

As a branch of glucose and glutamine metabolism, the hexosamine biosynthesis pathway (HBP) converts approximately 2%–5% glucose and a small fraction of glutamine to uridine diphosphate N-acetylglucosamine (UDP-GlcNAc), which plays an important role in the procession of classical glycosylation and O-GlcNAcylation (Figure 1A). We sought to determine whether HBP activation is associated with UBC development. By analyzing the genetic alterations of five HBP genes in UBC patients from TCGA-BLCA dataset, we found that *UAP1* gene was the major genetically altered HBP gene, with high frequent DNA amplification (∼16.0%) in UBCs. The rest of four HBP genes *GFPT1*, *GFPT2*, *GNPNAT1*, and *PGM3* displayed only 0.4%, 2.7%, 0.5%, and 2.2%, respectively (Figure 1B). This piece of evidence suggested that HBP might play an important role in UBC development.

Next, to explore the association between the expression levels of five HBP genes and UBC development, RNA-sequencing data were analyzed by using publicly available clinical genomics data repository. In TCGA-BLCA dataset, higher mRNA expression levels of *GFPT1*, *GNPNAT1*, and *PGM3* were detected in UBC patients than in normal bladder tissue, while lower *GFPT2* expression was observed in UBC, compared with normal bladder tissues (Figure 1C). Similarly, another independent GSE3167 dataset also displayed the increased mRNA expression levels of *GFPT1*, *PGM3*, and *UAP1* and the decreased *GFPT2* level in tumor tissues, while the data of *GNPNAT1* gene were not available in this dataset (Supplementary Figure S1).

To further investigate whether five HBP genes were involved in UBC progression, three datasets with tumor stage and tumor grade information were analyzed. In TCGA-BLCA dataset, higher mRNA levels of *GFPT1*, *GFPT2*, *PGM3*, and *UAP1* in Stage III–IV than those in Stage I–II were observed (Figure 1D). Meanwhile, we found all of five HBP genes were significantly elevated in high stages (T2–T4), compared with that in low stages (Ta + T1) in GSE13507 dataset (Figure 1E), and increased mRNA expression levels of *GFPT2*, *PGM3*, and *UAP1* in higher stages (T2–T4), compared with low stages (Ta + T1) in GSE32894 dataset, respectively (Supplementary Figure S1). Furthermore, we found that all five HBP genes overexpressed in high grade UBC patients in TCGA-BLCA and GSE13507 datasets, compared with low grade UBC patients, respectively (Figures 1F,G). In another independent cohort (GSE32894), the mRNA levels of *GFPT1*, *GFPT2*, *PGM3*, and *UAP1* were significantly elevated in high grade UBC samples, compared with low grade (Supplementary Figure S1). Altogether, the overexpression of HBP genes were frequently detected in UBC samples and tightly associated with UBC aggressiveness.

### The overexpression of hexosamine biosynthesis pathway genes predicted poor prognosis in urinary bladder cancer

**FIGURE 2:**
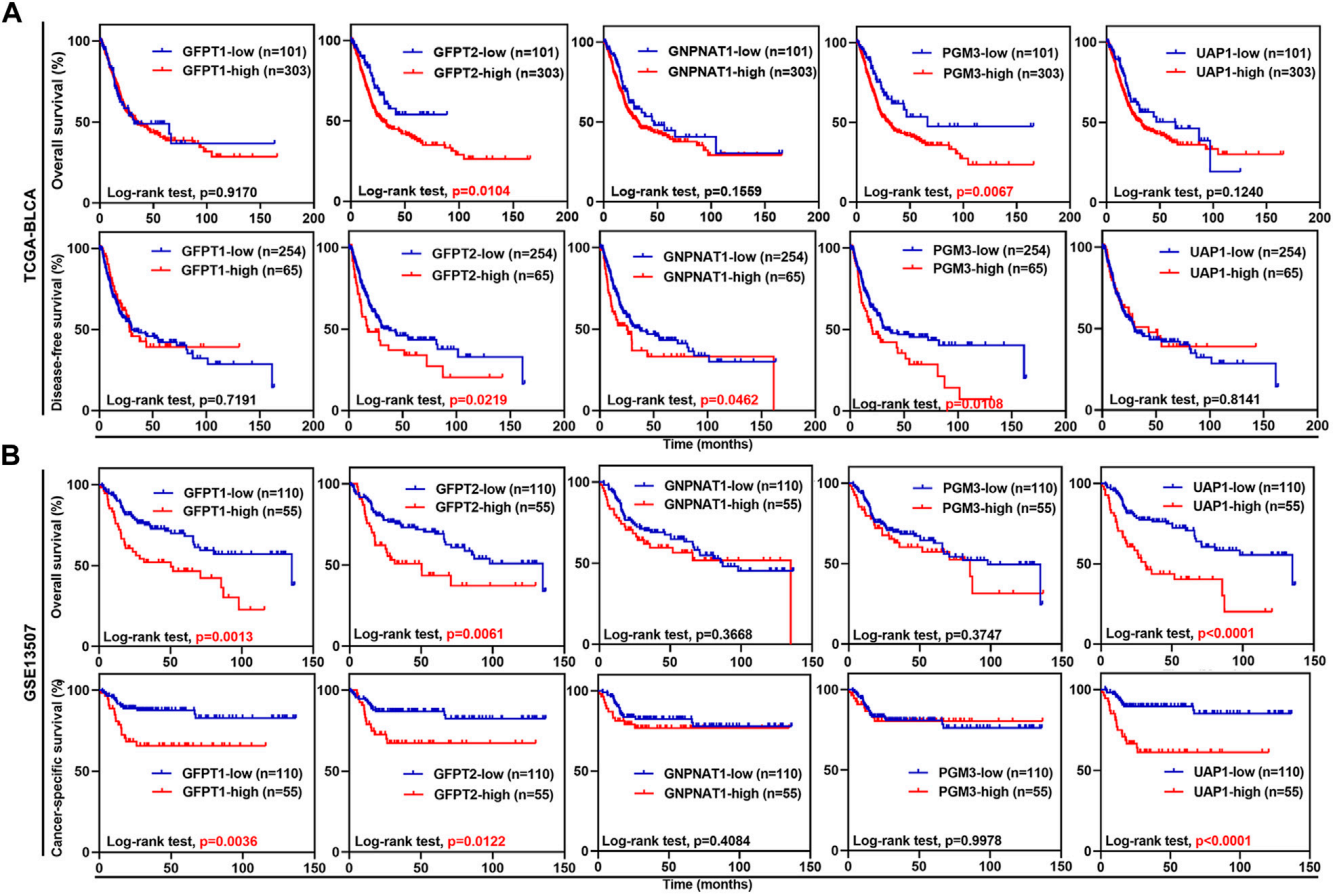
The associations between the expression levels of five HBP genes and clinical outcomes in UBC patients from TCGA-BLCA and GSE13507 datasets. **(A,B)** Kaplan-Meier plot of overall survival, disease-free survival or cancer-specific survival for UBC patients in TCGA-BLCA **(A)** and GSE13507 **(B)** datasets, stratified by the mRNA levels of the individual HBP gene. Survival probability was calculated using Log-rank test. *p* values < 0.05 were labelled in red.

Next, we examined whether the expression levels of the five HBP genes were associated with clinical outcomes. In TCGA-BLCA dataset, UBC patients with higher mRNA expression level of *GFPT2* and *PGM3* had significantly shorter OS time, while UBC patients with higher *GFPT2*, *GNPNAT1*, and *PGM3* mRNA levels had significantly shorter DFS duration (Figure 2A). In GSE13507 dataset, UBC patients with *GFPT1*, *GFPT2*, and *UAP1* overexpression experienced shorter OS and CSS durations, respectively (Figure 2B). Likewise, in GSE32894 dataset, *GFPT2*, *PGM3*, and *UAP1* overexpression predicted poor clinical outcomes in UBC patients, respectively (Supplementary Figure S2).

**FIGURE 3:**
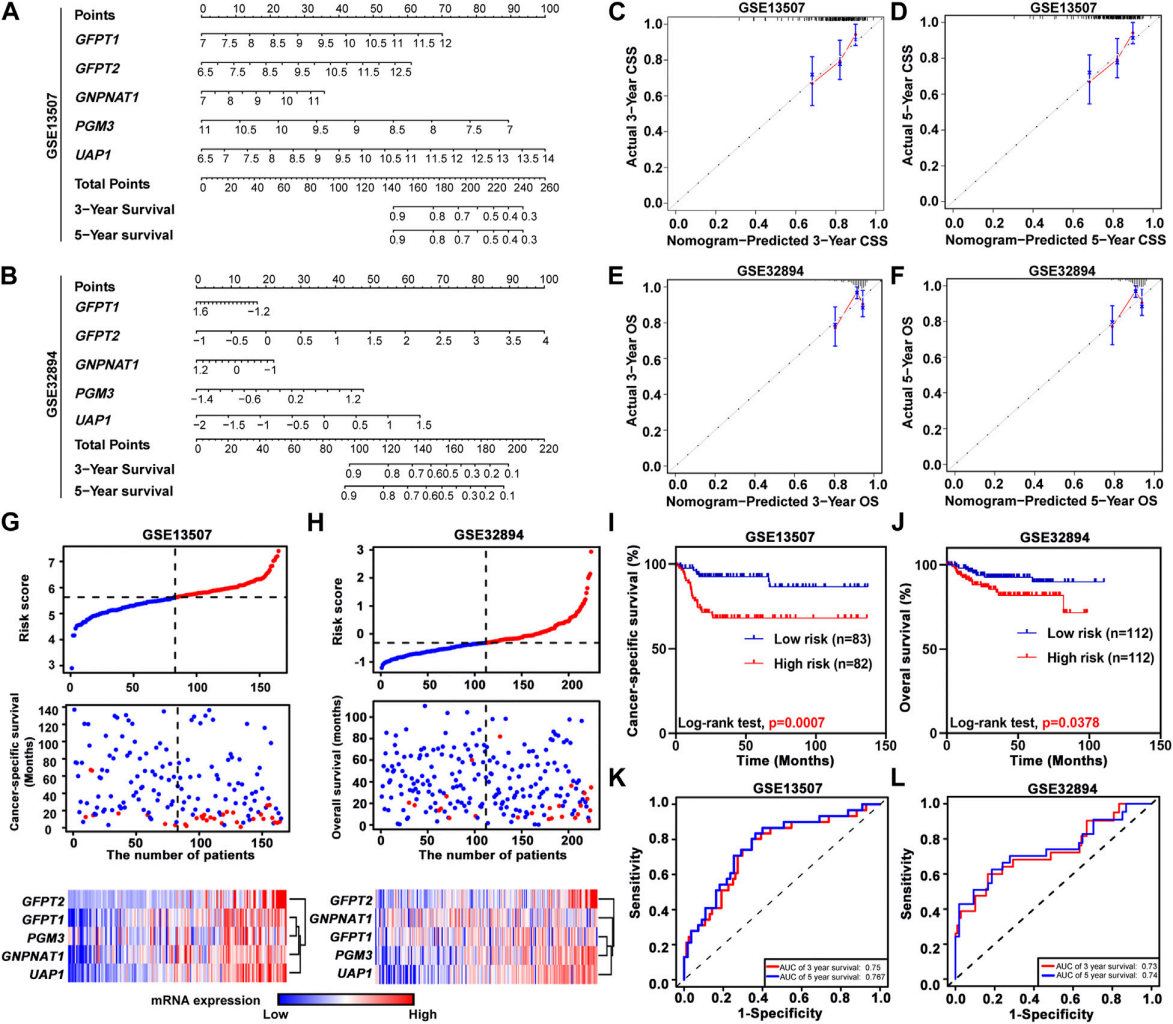
The relationship between prognostic value and the risk score of HBP in GSE13507 and GSE32894 datasets. **(A,B)** The nomogram was used to predict 3-, and 5-years cancer-specific survival CSS; **(A)** and overall survival OS; **(B)**. **(C–F)** The calibration curves of 3-, and 5-years CSS or OS of GSE13507 **(C,D)** and GSE32894 **(E,F)** datasets. The gray dots and gray lines represented the ideal prediction model, and the blue dots and red lines represented the observed models. **(G,H)** The distribution of risk scores, CSS-status and OS-status, and signature gene expression profiles in GSE13507 **(G)** and GSE32894 **(H)** datasets, respectively. **(I,J)** Kaplan-Meier plots of CSS of UBC patients in GSE13507 **(I)** and OS of UBC patients in GES32894 **(J)** datasets, stratified by the level of risk score, respectively. *p* values < 0.05 were labelled in red. **(K** and **L)** Time-dependent ROC curves for 3- (red lines) and 5-years (blue lines) survivals in GSE13507 **(K)** and GSE32894 **(L)** datasets.

To further study the prognostic value of HBP members, we incorporated the expression levels of these five genes by constructing predictive nomograms in GEO datasets (GSE13507 and GSE32894). With the C-index of 0.68 (GSE13507) and 0.70 (GSE32894), the nomograms showed a relatively good discrimination to predict 3- and 5-years CSS or OS of UBC patients (Figures 3A,B). In addition, the calibration curves were plotted. The observed models showed with red lines seemed relatively closed to the ideal prediction models with grey lines (Figures 3C–F). Furthermore, we calculated the risk scores based on these two GEO datasets. The distribution of risk scores, survival status, and signature gene expression profiles for five HBP genes were illustrated (Figures 3G,H). The results indicated that as the risk score of patients increased, the number of death events accumulated, and five HBP genes exhibited the increased expression. Kaplan-Meier survival curves indicated that UBC patients with high-risk scores had a significantly shorter CSS time, compared with low-risk patients in both GSE13507 and GSE32894 datasets (Figures 3I,J). In addition, both 3- and 5-years ROC curves of risk score showed a discriminative ability for 3-years and 5-years CSS in GSE13507 and OS in GSE32894 cohorts, respectively (Figures 3K,L).

### The hexosamine biosynthesis pathway signature was significantly associated with urinary bladder cancer aggressiveness

**FIGURE 4:**
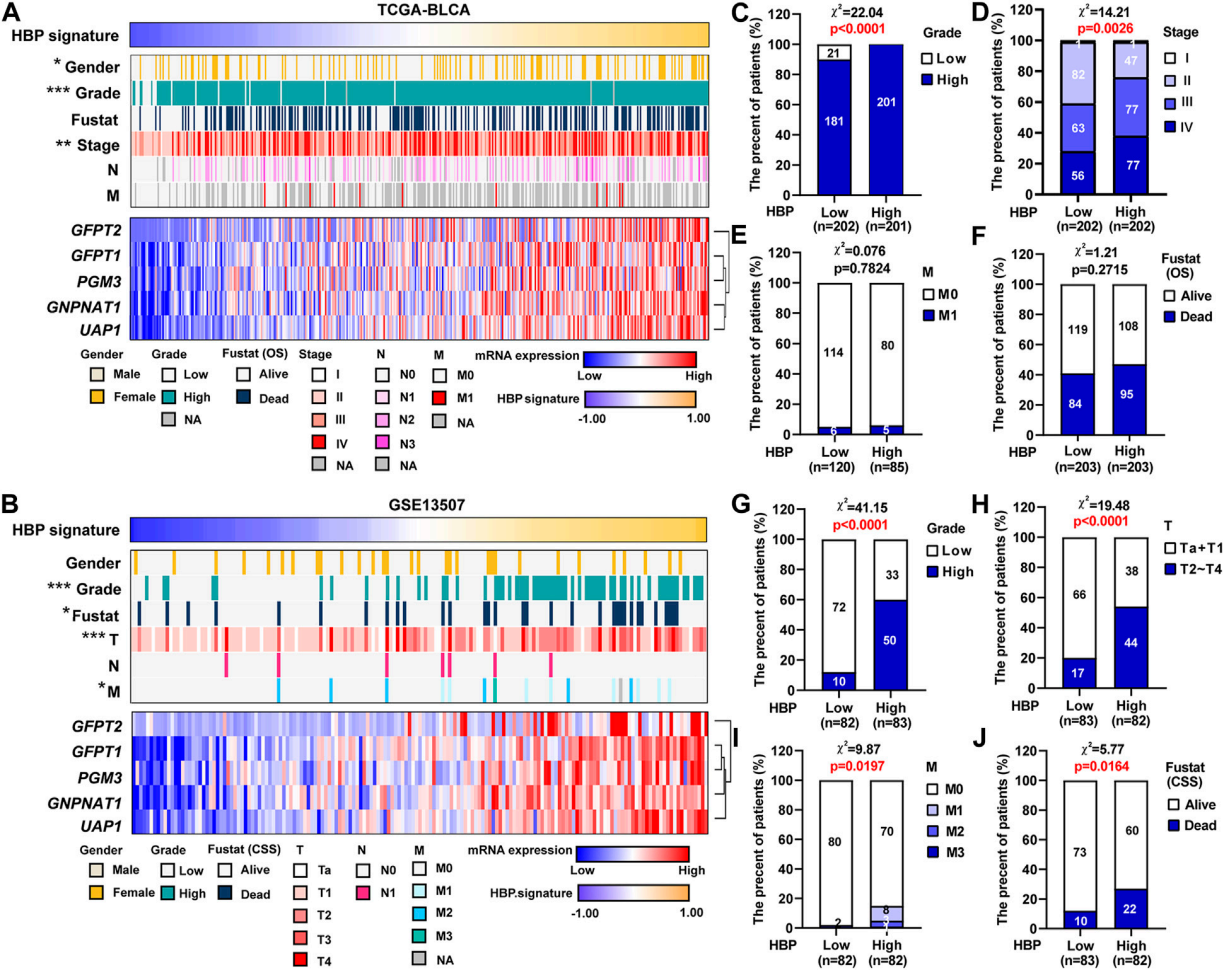
The associations between HBP signature level and clinicopathological parameters in UBC patients. **(A,B)** The heatmaps depicted the associations between HBP signature level and various clinicopathological parameters in TCGA-BLCA **(A)** and GSE13507 **(F)** datasets. **(C–J)** The differences between HBP signature and various clinicopathological parameters, including tumor grade **(C,G)**, tumor stage **(D,H)**, metastasis **(E,I)** and Fustat (OS or CSS) **(F,J)** in TCGA-BLCA **(C–F)** and GSE13507 **(G–J)** datasets. The Chi-square test was used. *p < 0.05, **p < 0.01, and ***p < 0.001. *p* values < 0.05 were labelled in red.

GSVA was performed in TCGA-BLCA and GSE13507 datasets. The samples were divided by the median of the HBP signature score. As shown in Supplementary Figure S3, HBP signature demonstrated the significant and strong associations with each five HBP genes in both TCGA-BLCA and GSE13507. This indicated that HBP signature represented the HBP genes well. Hence, the associations between HBP signature and other clinic variables were analyzed (Figures 4A,B). In terms of tumor stage and grade, the significant differences were observed in these two datasets (Figures 4C,D,G,H). In addition, we explored whether HBP groups were associated with metastasis stage (M stage) and survival status of UBC patients in TCGA-BLCA (Figures 4E,F) and GSE13507 (Figures 4I,J) datasets. Interestingly, we observed the high-HBP signature had the higher M stages and relatively higher number of patients died from UBC in GSE13507, compared with low-HBP signature (Figures 4I,J). Thus, our data suggested that the majority of UBC patients harboring higher HBP signature were associated with advanced-stage disease.

### GO and KEGG pathway analyses between high and low hexosamine biosynthesis pathway signature in urinary bladder cancer

**FIGURE 5:**
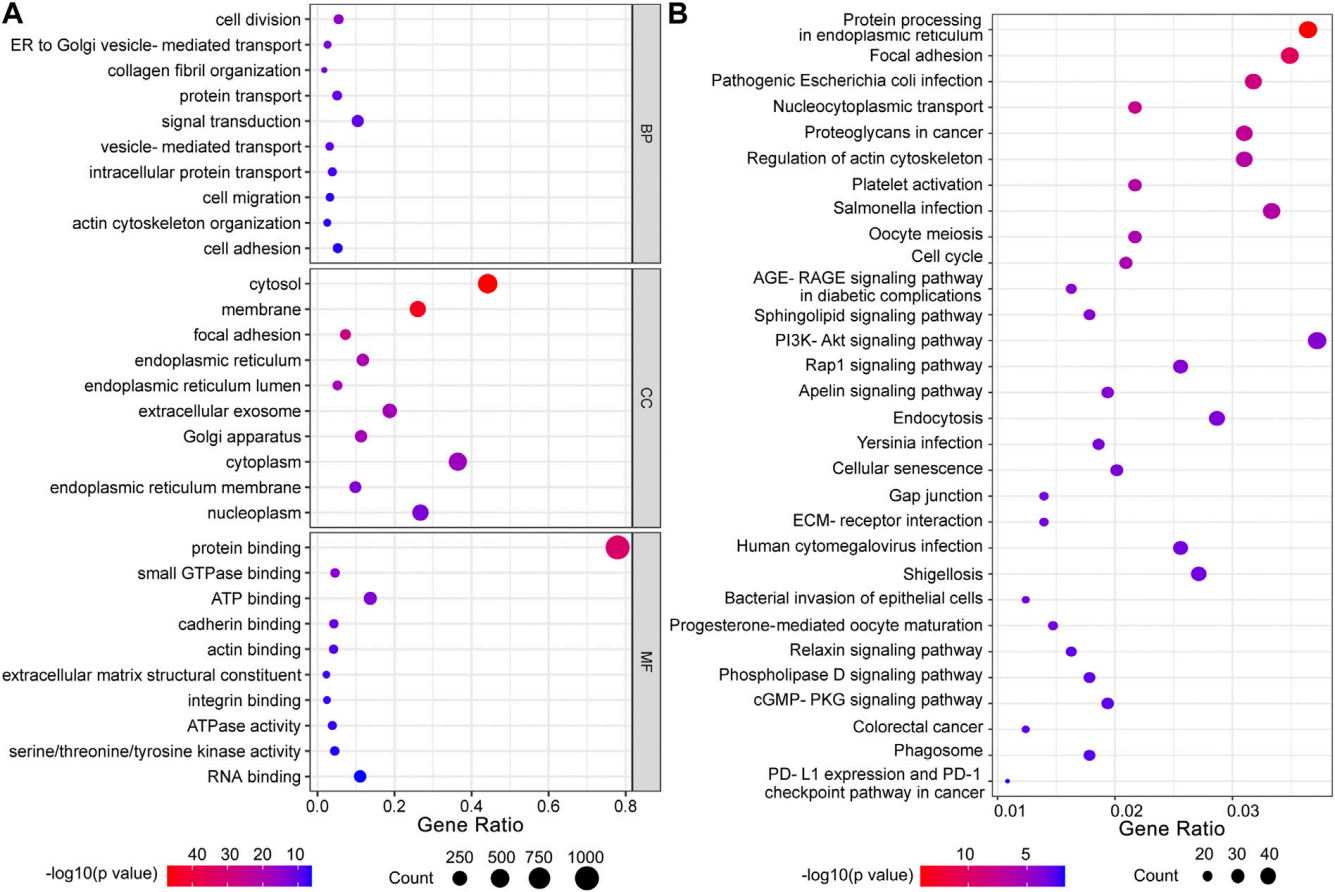
Functional enrichment analysis of co-expressed genes with HBP signature. **(A)** GO function analysis. BP: biological processes; CC: cellular components; MF: molecular function. **(B)** KEGG pathway analysis.

To investigate the potential biological function of HBP in UBC development, 1,293 genes co-expressed with HBP (Pearson r ≥ 0.25) were used for GO or KEGG pathway analysis. GO molecular function enrichment analysis showed that cell migration, cell adhesion and metabolic pathway were significantly enrichment (Figure 5A). KEGG pathway analysis revealed that immunoglobulin-mediated immune responses, such as pathogenic *Escherichia coli* infection, *Salmonella* infection, *Yersinia* infection, PD-L1 expression and PD-1 checkpoint pathway in cancer were remarkably enriched (Figure 5B).

### The hexosamine biosynthesis pathway signature in urinary bladder cancer was closely associated with immune infiltration

**FIGURE 6:**
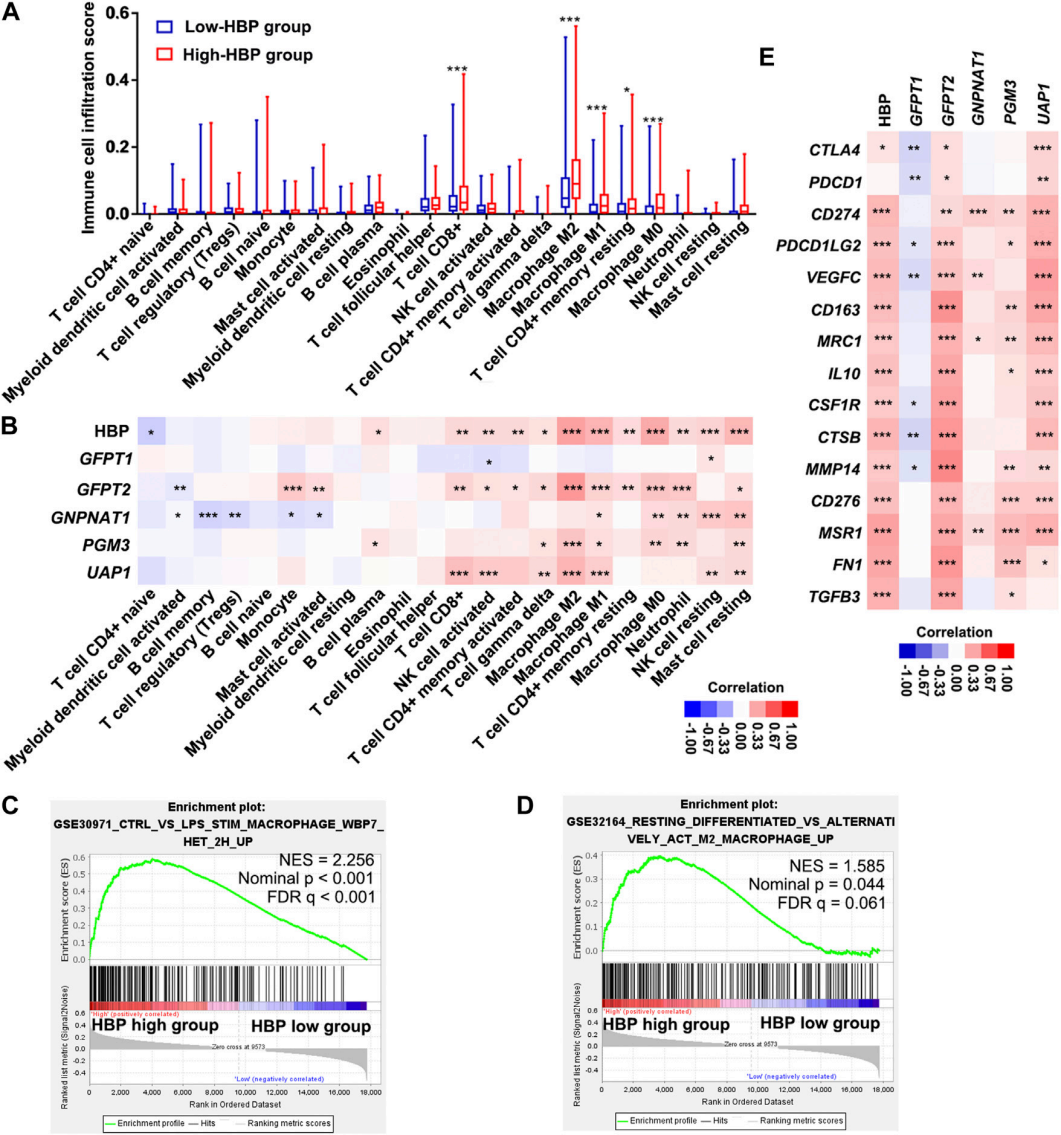
HBP was positively correlated with the infiltration of macrophages in TCGA-BLCA dataset. **(A)** The infiltrating estimation fraction of immune cell in low- and high-HBP signature groups. **(B)** Heatmap for the associations between the infiltrating estimation fraction and HBP score or mRNA level of each HBP member in different immune cell types. **(C,D)** The associations between HBP signature and macrophage signaling **(C)** or M2 macrophage signaling **(D)** in TCGA-BLCA dataset by GSEA. In the enrichment plot, genes were ranked by signal/noise ratio according to their differential scores between low- and high-HBP signatures. **(E)** Heatmap for the associations between M2 macrophage-associated genes expression and HBP score or mRNA level of each HBP member. *p < 0.05, **p < 0.01, and ***p < 0.001.

We analyzed the infiltrating estimation fraction of immune cells, which was performed by CIBERSORT-ABS algorithm in high or low HBP signature score groups. As shown in Figure 6A, macrophages, including M0, M1, and M2, were significantly enriched in HBP-high group. Consistently, HBP signature score was significantly associated with macrophage infiltrating fraction (Figure 6B). Similar to HBP signature, *GFPT2*, *PGM3*, and *UAP1* mRNA levels were also positively correlated with macrophage M2 infiltrating fraction, respectively (Figure 6B). To analyze the potential function of HBP in immunoregulation, GSEA were used to evaluate the relationship between HBP and immunologic signatures in C7 immunologic signature gene sets (V7.5). Likewise, macrophage associated signature was highlighted in top 10 significance immunologic signatures (Supplementary Table S2, Figure 6C). Given that PD-L1/PD-1 pathway was enriched in these co-expression genes with HBP, we assessed the association between HBP signature and M2 macrophage signature. As shown in Figure 6D, GSEA confirmed that M2 macrophage genes were also enriched in HBP-high groups. In addition, HBP signature and three HBP genes (*GFPT2*, *PGM3*, and *UAP1*) were significantly associated with M2 macrophage-associated gene expression (Kfoury et al., 2021; Figure 6E). This hinted that HBP may be associated with the infiltration or polarization of M2 macrophage.

## Discussion

Glucose metabolism is frequently reprogrammed in cancer cells, due to the alterations of oncogenes and tumor suppressor genes (Akella et al., 2019; Lam et al., 2021). As a branch of the glycolytic pathway, HBP incorporates several major metabolites, such as glucose, glutamine, acetyl-CoA, and UTP to produce UDP-GlcNAc. Hence, HBP is one of the key pathways that link the metabolic sensing and cellular signalings. In this study, we comprehensively investigated the clinical relevance of five HBP genes in four independent public UBC datasets (TCGA-BLCA, GSE13507, GSE32894, and GSE3167), containing 948 UBC samples with 100 normal samples in total. We not only found that the majority of HBP genes, but also HBP-high-risk score group were positively associated with UBC aggressiveness and poor clinical outcomes in UBC patients. Interestingly, high-HBP signature group displayed a significant association with immune infiltration in UBC.

Recent cumulating evidence suggested that HBP may play a tumor-promoting role in the development of multiple cancer types by studying the individual HBP genes. As one of the first rate-limiting enzymes in HBP, GFPT1 overexpression is associated with a poor prognosis in prostate cancer (Itkonen et al., 2013), colon cancer (Vasconcelos-Dos-Santos et al., 2017), and pancreatic cancer (Yang et al., 2016). GFAT1 expression can be induced by extracellular stimuli, such as high glucose concentration and pro-inflammatory chemokine IL-8, respectively. GFPT1 overexpression in return increases O-GlcNAcylation in cancer cells, and consequently promotes cancer cell proliferation, invasion and cancer stemness (Phoomak et al., 2017; Shimizu and Tanaka 2019). The expression of GFPT2, an isoenzyme of GFPT1, is specifically induced in KRAS/LKB1 combined mutant lung cancer cells, and promotes tumorigenicity by enhancing glucose influx into HBP. UAP1, the second rate-limiting enzyme in the final step of HBP, is also upregulated in prostate cancer and UBC (Itkonen et al., 2015; Puttamallesh et al., 2020). The UAP1 activity can be inhibited by Fbxl17 through blocking the phosphorylation status of UAP1 in breast cancer (Mason et al., 2020). In our study, we found that *UAP1* gene is amplified in ∼16% of UBC patients in TCGA-BLCA dataset, suggesting another molecular mechanism for the increased UAP1 activity during cancer development. Though relatively fewer studies on another two HBP genes, *GNPNAT1* and *PGM3*, the overexpression of GNPNAT1 and PGM3 was reported in lung adenocarcinoma and prostate cancer, respectively (Munkley et al., 2016; Liu et al., 2021).

In addition, the development of liquid chromatography-mass spectrometry (LC-MS) leads to examine the activation of HBP as a whole functional pathway, instead of investigating individual HBP gene expression. For example, the metabolomics analysis on hepatic cancer cells revealed that CD133^+^ hepatic cancer stem cells produced remarkable and significant amounts of HBP metabolites, such as UDP-GlcNAc, compared with CD133^-^ non-cancer stem cells (Lin et al., 2016). The enhanced HBP flux was reported in primary breast cancer cells derived from MMTV-Neu and MMTV-PyVT mouse models, compared with normal mouse mammary epithelial cells (Chokchaitaweesuk et al., 2019). Interestingly, an amino sugar metabolism (known as HBP) signature was specifically elevated in *Kras/Lkb1* co-mutated mouse lung tumors, compared with both *Kras* mutated and *Kras/p53* co-mutated lung tumors by metabolomics analysis, which was further confirmed by the investigation on the HBP flux using a stable isotope-assisted tracing method (Kim et al., 2020). Overall, the HBP is frequently dysregulated in cancer development.

However, the activation of HBP flux may also play a tumor-suppressive role under certain circumstances. GFAT1 is negatively associated with gastric cancer cell invasion and it predicts a favorable prognosis in gastric cancer (Duan et al., 2016). Besides, the mRNA and protein levels of GNPNAT1 and UAP1, as well as the ratio of the intermediate metabolites (GlcNAc-6-P/GlcN-6-P), are significantly elevated in prostate cancers, compared with benign prostatic hyperplasia. However, in the castration-resistant prostate cancer (CRPC) both GNPNAT1 and UAP1 are significantly downregulated. Notably, the treatment of CRPC cells with the HBP metabolite UDP-GlcNAc displayed tumor inhibitory effects and also sensitized CRPC cells to enzalutamide *in vitro* (Kaushik et al., 2016). These pieces of evidence indicated that HBP activation is cancer cell-context dependent and cancer cells may adapt to various microenvironments or therapies through metabolic rewiring.

Tumor microenvironment (TME) is a complex system that includes direct interactions between cancer cells and immune cells (Zheng et al., 2020). Recently, GFPT1 was found to be overexpressed in pancreatic ductal adenocarcinoma (PDAC) and promoted cancer stemness and metastasis. Once knockdown of GFPT1 or abrogation of its activity by a glutamine analog (6-diazo-5-oxo-l-norleucine, DON) in PDAC cells, these cells in co-cultured with cancer-associated fibroblasts reduced the secretion of tumor-promoting chemokine IL-27, as well as the production of hyaluronan, whose synthesis needs UDP-GlcNAc as one of the primary substrates (Sharma et al., 2020). Since the presence of hyaluronan was regarded as a major reason for the immune evasion of PDAC (McCarthy et al., 2018), abrogation of GFPT1 by DON increased the anti-tumor CD68^+^ macrophage and cytotoxic T cells, eventually sensitized anti-PD-1 immunotherapy effects. Our data revealed that HBP-high signature was significantly and positively associated with M2 macrophages and CD8^+^ T cells as well as the expression of CD274/PD-L1. The infiltration of immunosuppressive macrophages and the high expression levels of PD-L1 will induce T cell immune exhaustion (Braun et al., 2021; Kersten et al., 2022). Therefore, it is intriguing to examine whether the HBP activation is a promising therapeutic target in UBC patients for immunotherapy.

In order to overview the clinical significance of HBP genes as a whole, instead of an individual gene, we carried out the comprehensive analysis on five HBP genes in various public UBC cohorts. Notably, our data indicated not only the poor clinical outcomes in HBP-high-risk score group, but also the strong association of HBP signature with immune infiltration. Our *in silico* study suggested that targeting HBP enzymes will be a potentially feasible approach to treat UBC cells in future. Actually, targeting GFPT2 by azaserine and targeting PGM3 by FR054 can specifically inhibit KRAS/LKB1 combined mutant lung adenocarcinoma cells, respectively (Kim et al., 2020; Lee et al., 2022). In addition, targeting GFPT1 by DON blocked the PCK1-loss induced O-GlcNAclyation and liver cancer aggressiveness (Xiang et al., 2021), and enhanced anti-PD-1 immunotherapy effects in pancreatic cancer (Sharma et al., 2020). It would be interesting to functionally examine whether targeting the key enzymes in the HBP by RNA interfering, as well as small molecular inhibitors, could reduce UBC cell survival and/or sensitize cancer cells to immunotherapy *in vitro* and *in vivo*. Therefore, it encourages us to investigate whether targeting HBP activation will benefit UBC patients in future.

## Data Availability

The datasets presented in this study can be found in online repositories. The names of the repository/repositories and accession number(s) can be found in the article/Supplementary Material.
