# Supplementary material for: Identification of hexosamine biosynthesis pathway as a novel prognostic signature and its correlation with immune infiltration in bladder cancer
Source: Front Mol Biosci. 2022 Sep 8;9:1009168. doi: 10.3389/fmolb.2022.1009168 (PMC9493074; doi:10.3389/fmolb.2022.1009168)
Supplement: Supplementary file 1 [file Table1.DOCX]

Supplementary Material

**Identification of hexosamine biosynthesis pathway as a novel prognostic signature and its correlation with immune infiltration in bladder cancer**

Yangyan Cui^1,†^, Hanyi Feng^2,3,†^, Jiakuan Liu^4,5,†^, Jiajun Wu^4^, Rujian Zhu^5,*^, Ruimin Huang^2,3,*^, Jun Yan^4,5,6,*^

^1^ Model Animal Research Center, Nanjing University,12 Xuefu Road, Nanjing 210061, Jiangsu, China

^2^ School of Chinese Materia Medica, Nanjing University of Chinese Medicine, 138 Xianlin Avenue, Nanjing 210023, Jiangsu, China

^3^ Shanghai Institute of Materia Medica, Chinese Academy of Sciences, 555 Zuchongzhi Road, Shanghai 201203, China

^4^ Department of Laboratory Animal Science, Fudan University, 130 Dong’an Road, Shanghai 200032, China

^5^ Department of Urology, Shanghai Pudong Hospital, Fudan University Pudong Medical Center, 2800 Gongwei Road, Shanghai 201399, China

^6^ MOE Key Laboratory of Model Animals for Disease Study, Model Animal Research Center of Nanjing University, Nanjing 210061, Jiangsu, China.

**†**These authors have contributed equally to this work.

*** Correspondence:**Rujian Zhu, M.D.

[20667@shpdh.org](mailto:20667@shpdh.org)

Ruimin Huang, Ph.D.
[rmhuang@simm.ac.cn](mailto:rmhuang@simm.ac.cn)

Jun Yan, Ph.D.
[yan_jun@fudan.edu.cn](mailto:yan_jun@fudan.edu.cn)

## Supplementary Figures


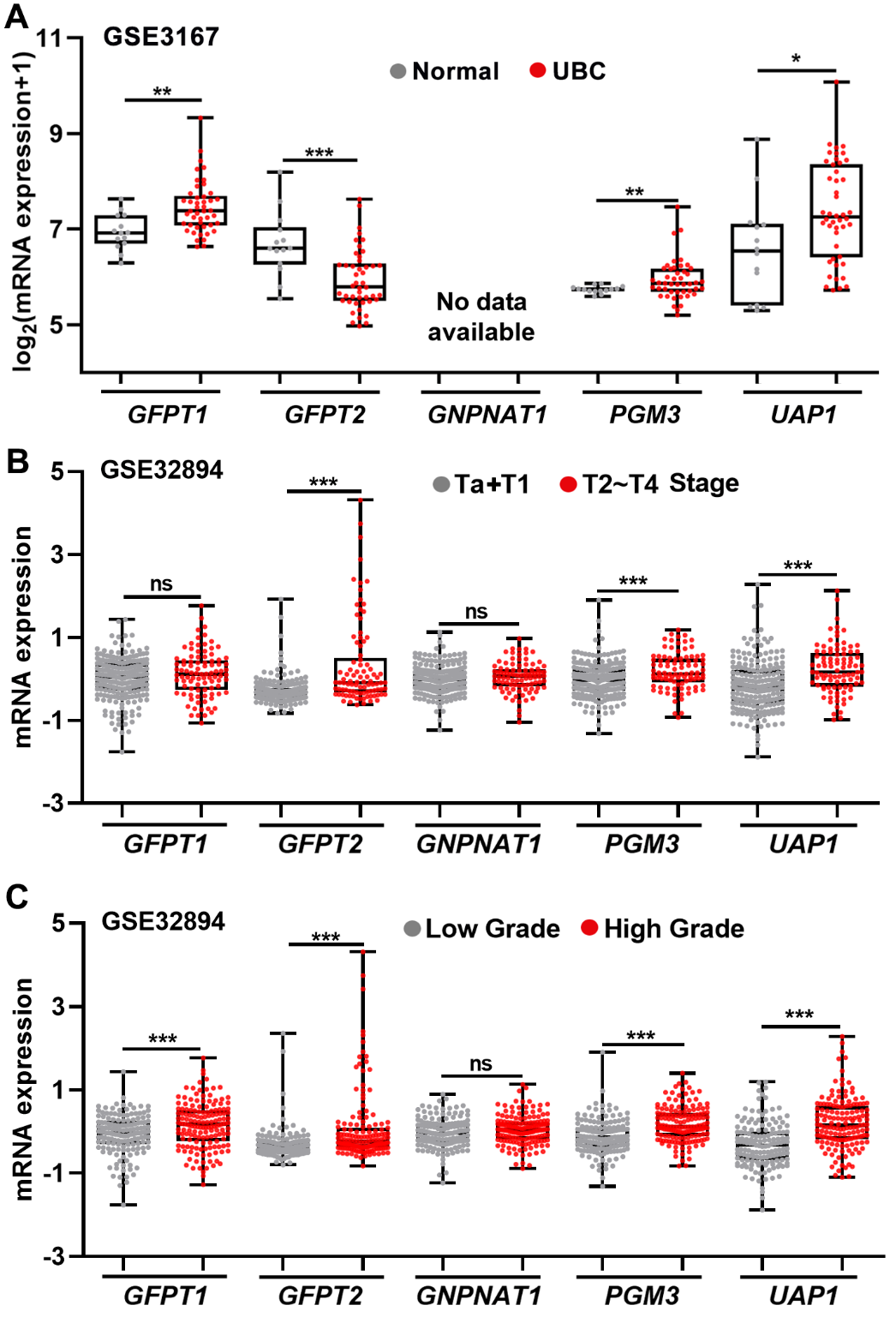


**Supplementary Figure 1 |** The relationships between gene expression levels of five HBP genes and clinicopathological factors in UBC patients. **(A)** The mRNA levels of five HBP genes in normal bladder tissues (n = 14) and UBC tissues (n = 46) in GSE3167 dataset. **(B)** The mRNA levels of five HBP genes in low stage (Ta+T1, n = 213) and high stage (T2~T4, n = 93) of UBCs from GSE32894. **(C)** The mRNA levels of five HBP genes in low grade (n = 151) and high grade (n = 155) of UBCs from GSE13507 dataset. *p *<* 0.05, **p *<* 0.01, ***p *<* 0.001, and ns, non-significant.


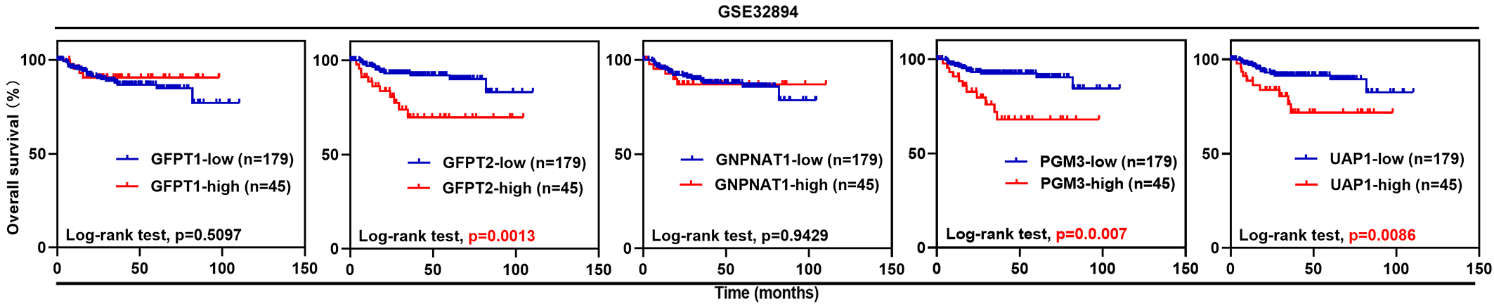


**Supplementary Figure 2 |** Kaplan-Meier plots of overall survival of UBC patients in GSE32894 datasets, stratified by mRNA levels of each HBP member, respectively. Survival probability was calculated using Log-rank test. p values < 0.05 were labelled in red.


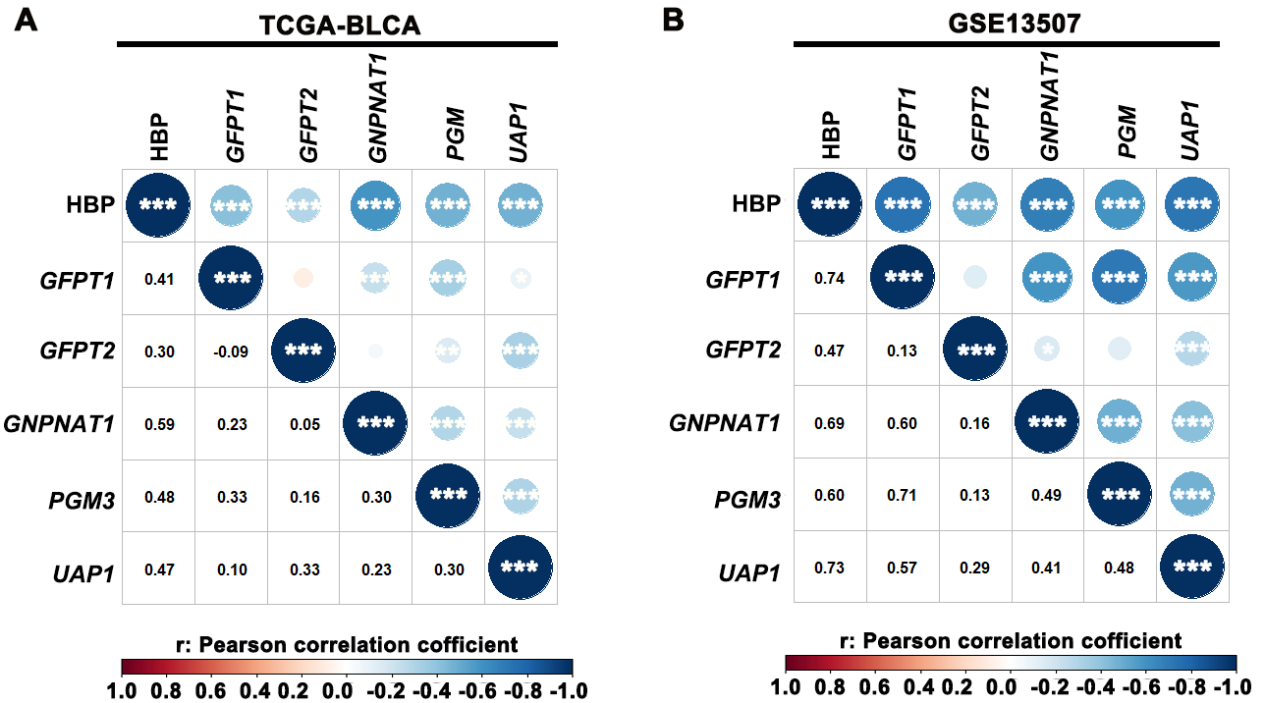


**Supplementary Figure 3 |** The correlations between HBP signature and each HBP member (*GFPT1*, *GFPT2*, *GNPNAT1*, *PGM3* and *UAP1*) in TCGA-BLCA (**A**) and GSE13507 (**B**) datasets. The circle in blue, positive correlation; the circle in red, negative correlation. The sizes of circles and the number in boxes indicated the Pearson correlation coefficient. * p *<* 0.05, **p *<* 0.01, ***p *<* 0.001.

| **Supplementary table 1.** **The list of 1,293 genes positively associated with HBP signature identified by Pearson's correlation** | | | | | | |
| --- | --- | --- | --- | --- | --- | --- |
| **Gene symbol or pathway** | **Pearson correlation** | **p value** |  | **Gene symbol or pathway** | **Pearson correlation** | **p value** |
| **HBP** | 1 | 0 |  | *KIF14* | 0.298 | 9.51E-10 |
| *CALU* | 0.614 | 1.90E-43 |  | *DLAT* | 0.298 | 9.54E-10 |
| *GNPNAT1* | 0.592 | 9.39E-40 |  | *NOMO2* | 0.297 | 9.70E-10 |
| *SEC23A* | 0.518 | 3.28E-29 |  | *MED8* | 0.297 | 9.81E-10 |
| *PDIA6* | 0.516 | 5.41E-29 |  | *MORC4* | 0.297 | 9.87E-10 |
| *SSR3* | 0.507 | 6.26E-28 |  | *FPR1* | 0.297 | 1.00E-09 |
| *MCFD2* | 0.506 | 8.51E-28 |  | *TGFB3* | 0.297 | 1.01E-09 |
| *SURF4* | 0.504 | 1.71E-27 |  | *CDC42EP3* | 0.297 | 1.02E-09 |
| *CDV3* | 0.493 | 2.93E-26 |  | *NOTCH2* | 0.297 | 1.04E-09 |
| *HSPA5* | 0.493 | 2.99E-26 |  | *SPART* | 0.297 | 1.08E-09 |
| *LRRC59* | 0.493 | 3.04E-26 |  | *ZW10* | 0.297 | 1.08E-09 |
| *SEC24D* | 0.489 | 7.66E-26 |  | *FAM219A* | 0.296 | 1.11E-09 |
| *ITGB1* | 0.480 | 7.93E-25 |  | *HPS5* | 0.296 | 1.12E-09 |
| *GOLT1B* | 0.479 | 9.96E-25 |  | *C12orf4* | 0.296 | 1.16E-09 |
| *PGM3* | 0.475 | 3.00E-24 |  | *SMIM13* | 0.296 | 1.16E-09 |
| *UAP1* | 0.473 | 4.92E-24 |  | *MASTL* | 0.296 | 1.17E-09 |
| *TPM3* | 0.471 | 7.77E-24 |  | *TBL2* | 0.296 | 1.17E-09 |
| *RECQL* | 0.471 | 8.75E-24 |  | *NEK7* | 0.296 | 1.19E-09 |
| *SLC39A14* | 0.464 | 4.80E-23 |  | *LIPG* | 0.296 | 1.20E-09 |
| *ARF4* | 0.463 | 5.29E-23 |  | *ZRANB1* | 0.296 | 1.21E-09 |
| *KDELR2* | 0.461 | 9.88E-23 |  | *GALNT2* | 0.296 | 1.23E-09 |
| *UBQLN1* | 0.460 | 1.08E-22 |  | *GFPT2* | 0.296 | 1.23E-09 |
| *IARS1* | 0.455 | 4.08E-22 |  | *UHRF1BP1L* | 0.296 | 1.25E-09 |
| *ARCN1* | 0.453 | 5.50E-22 |  | *PSMG1* | 0.296 | 1.25E-09 |
| *ACTR2* | 0.449 | 1.48E-21 |  | *CCNK* | 0.296 | 1.26E-09 |
| *SLC31A1* | 0.449 | 1.53E-21 |  | *STT3A* | 0.295 | 1.27E-09 |
| *CLIC4* | 0.449 | 1.71E-21 |  | *SMAP1* | 0.295 | 1.28E-09 |
| *HSPA13* | 0.448 | 1.84E-21 |  | *THEMIS2* | 0.295 | 1.28E-09 |
| *PDIA5* | 0.448 | 1.97E-21 |  | *CALHM5* | 0.295 | 1.30E-09 |
| *LATS2* | 0.448 | 2.04E-21 |  | *TMEM241* | 0.295 | 1.32E-09 |
| *ALDH1L2* | 0.448 | 2.12E-21 |  | *MFSD1* | 0.295 | 1.33E-09 |
| *SGMS2* | 0.444 | 4.58E-21 |  | *B3GNT5* | 0.295 | 1.36E-09 |
| *MTHFD2* | 0.444 | 4.72E-21 |  | *NCAPH* | 0.295 | 1.37E-09 |
| *SEC24A* | 0.444 | 4.77E-21 |  | *DDR2* | 0.295 | 1.37E-09 |
| *ACBD3* | 0.444 | 4.87E-21 |  | *JAZF1* | 0.295 | 1.37E-09 |
| *SEC31A* | 0.444 | 5.00E-21 |  | *ATP10A* | 0.295 | 1.38E-09 |
| *RAB31* | 0.443 | 5.69E-21 |  | *S100A10* | 0.295 | 1.38E-09 |
| *KDELR3* | 0.443 | 5.76E-21 |  | *DCBLD1* | 0.295 | 1.40E-09 |
| *EPRS1* | 0.443 | 5.99E-21 |  | *FBXO45* | 0.295 | 1.43E-09 |
| *YME1L1* | 0.441 | 8.51E-21 |  | *ADAMTS6* | 0.295 | 1.43E-09 |
| *ABL2* | 0.441 | 1.00E-20 |  | *USP37* | 0.295 | 1.43E-09 |
| *ANXA5* | 0.439 | 1.43E-20 |  | *C3AR1* | 0.295 | 1.44E-09 |
| *RSU1* | 0.437 | 2.37E-20 |  | *CCN1* | 0.294 | 1.45E-09 |
| *PLS3* | 0.435 | 3.62E-20 |  | *RUBCN* | 0.294 | 1.48E-09 |
| *MPZL1* | 0.435 | 3.93E-20 |  | *TTL* | 0.294 | 1.50E-09 |
| *CEBPZ* | 0.434 | 4.27E-20 |  | *PRR16* | 0.294 | 1.51E-09 |
| *TAF13* | 0.433 | 5.83E-20 |  | *RALA* | 0.294 | 1.52E-09 |
| *RAB11FIP5* | 0.433 | 5.99E-20 |  | *RNF144A* | 0.294 | 1.53E-09 |
| *STYX* | 0.432 | 6.64E-20 |  | *PSAT1* | 0.294 | 1.54E-09 |
| *RASAL2* | 0.432 | 7.34E-20 |  | *LUZP1* | 0.294 | 1.55E-09 |
| *DCLRE1B* | 0.430 | 9.62E-20 |  | *VPS26A* | 0.294 | 1.55E-09 |
| *AIDA* | 0.429 | 1.21E-19 |  | *DEGS1* | 0.294 | 1.58E-09 |
| *ASAP1* | 0.428 | 1.62E-19 |  | *POLE3* | 0.294 | 1.59E-09 |
| *ASAP2* | 0.423 | 4.78E-19 |  | *DARS2* | 0.294 | 1.63E-09 |
| *ANLN* | 0.421 | 7.20E-19 |  | *TUBA1B* | 0.294 | 1.64E-09 |
| *COLGALT1* | 0.419 | 9.99E-19 |  | *AMD1* | 0.293 | 1.67E-09 |
| *DDX21* | 0.419 | 1.19E-18 |  | *NCKAP1* | 0.293 | 1.69E-09 |
| *CORO1C* | 0.418 | 1.43E-18 |  | *SDHC* | 0.293 | 1.72E-09 |
| *EHBP1* | 0.417 | 1.74E-18 |  | *CD109* | 0.293 | 1.72E-09 |
| *NRAS* | 0.416 | 1.89E-18 |  | *VANGL1* | 0.293 | 1.76E-09 |
| *VRK2* | 0.416 | 1.95E-18 |  | *BCAT1* | 0.293 | 1.76E-09 |
| *PNO1* | 0.416 | 1.98E-18 |  | *IGF2R* | 0.293 | 1.77E-09 |
| *FAP* | 0.416 | 2.02E-18 |  | *SEC24C* | 0.293 | 1.77E-09 |
| *AFAP1* | 0.416 | 2.09E-18 |  | *CBL* | 0.293 | 1.80E-09 |
| *VKORC1L1* | 0.414 | 2.99E-18 |  | *ADPRH* | 0.293 | 1.83E-09 |
| *GFPT1* | 0.414 | 3.29E-18 |  | *ZEB2* | 0.292 | 1.89E-09 |
| *SRP72* | 0.413 | 3.79E-18 |  | *SGCB* | 0.292 | 1.90E-09 |
| *PTPN12* | 0.413 | 3.80E-18 |  | *SLC8A1* | 0.292 | 1.91E-09 |
| *YWHAG* | 0.410 | 7.01E-18 |  | *TOR1A* | 0.292 | 1.92E-09 |
| *USP14* | 0.409 | 8.20E-18 |  | *CYP51A1* | 0.292 | 1.93E-09 |
| *SAV1* | 0.409 | 8.47E-18 |  | *RETSAT* | 0.292 | 1.95E-09 |
| *HDLBP* | 0.409 | 8.50E-18 |  | *CSMD2* | 0.292 | 1.97E-09 |
| *CNIH1* | 0.408 | 9.88E-18 |  | *SMC1A* | 0.292 | 1.99E-09 |
| *SLC12A8* | 0.408 | 1.01E-17 |  | *TGFBR1* | 0.292 | 2.02E-09 |
| *STK3* | 0.407 | 1.37E-17 |  | *AC069544.2* | 0.292 | 2.06E-09 |
| *SETD7* | 0.406 | 1.43E-17 |  | *MAD2L1* | 0.292 | 2.06E-09 |
| *MBTPS2* | 0.406 | 1.43E-17 |  | *RPN2* | 0.292 | 2.07E-09 |
| *PSMD2* | 0.406 | 1.47E-17 |  | *DNAJB5* | 0.292 | 2.08E-09 |
| *PALM2AKAP2* | 0.406 | 1.51E-17 |  | *HIPK3* | 0.292 | 2.10E-09 |
| *GUCY1A1* | 0.406 | 1.56E-17 |  | *CDC20* | 0.292 | 2.13E-09 |
| *WASHC5* | 0.406 | 1.64E-17 |  | *CCL11* | 0.291 | 2.16E-09 |
| *RAD23B* | 0.402 | 3.42E-17 |  | *UBE3C* | 0.291 | 2.17E-09 |
| *TNFAIP6* | 0.401 | 4.20E-17 |  | *TUBB6* | 0.291 | 2.20E-09 |
| *COPB1* | 0.401 | 4.49E-17 |  | *ATP11B* | 0.291 | 2.20E-09 |
| *RAB1A* | 0.401 | 4.49E-17 |  | *MSH2* | 0.291 | 2.21E-09 |
| *NEK6* | 0.400 | 4.80E-17 |  | *ARHGAP24* | 0.291 | 2.21E-09 |
| *GNB4* | 0.399 | 6.56E-17 |  | *CAV1* | 0.291 | 2.22E-09 |
| *SND1* | 0.399 | 6.63E-17 |  | *DPYD* | 0.291 | 2.24E-09 |
| *FKBP9* | 0.398 | 6.96E-17 |  | *TNPO1* | 0.291 | 2.26E-09 |
| *KPNA4* | 0.398 | 7.78E-17 |  | *ISCA1* | 0.291 | 2.36E-09 |
| *STRAP* | 0.398 | 8.05E-17 |  | *ADGRE5* | 0.291 | 2.38E-09 |
| *ACTN1* | 0.397 | 8.11E-17 |  | *TRIM44* | 0.291 | 2.39E-09 |
| *PRXL2C* | 0.397 | 8.44E-17 |  | *LRP1* | 0.291 | 2.40E-09 |
| *ARHGAP21* | 0.396 | 1.00E-16 |  | *RAD21* | 0.291 | 2.44E-09 |
| *HSPA8* | 0.395 | 1.19E-16 |  | *TRIP13* | 0.291 | 2.44E-09 |
| *C2orf69* | 0.395 | 1.20E-16 |  | *GALNT3* | 0.290 | 2.50E-09 |
| *MMGT1* | 0.395 | 1.24E-16 |  | *CHAC2* | 0.290 | 2.52E-09 |
| *COPB2* | 0.394 | 1.46E-16 |  | *CAPN2* | 0.290 | 2.56E-09 |
| *OSMR* | 0.394 | 1.64E-16 |  | *FLNA* | 0.290 | 2.58E-09 |
| *PLIN3* | 0.393 | 1.83E-16 |  | *EXT2* | 0.290 | 2.67E-09 |
| *ATL3* | 0.392 | 2.12E-16 |  | *PRR11* | 0.290 | 2.75E-09 |
| *ATF6* | 0.392 | 2.22E-16 |  | *ECE1* | 0.290 | 2.75E-09 |
| *ECT2* | 0.391 | 2.65E-16 |  | *NID1* | 0.290 | 2.76E-09 |
| *SH3KBP1* | 0.391 | 2.72E-16 |  | *EHD1* | 0.290 | 2.77E-09 |
| *CKAP2L* | 0.390 | 3.13E-16 |  | *CRTAP* | 0.289 | 2.85E-09 |
| *LMAN1* | 0.390 | 3.26E-16 |  | *GULP1* | 0.289 | 2.88E-09 |
| *CSGALNACT2* | 0.390 | 3.27E-16 |  | *HSD17B12* | 0.289 | 2.96E-09 |
| *DDX18* | 0.390 | 3.27E-16 |  | *AGTPBP1* | 0.289 | 3.05E-09 |
| *FAM114A1* | 0.390 | 3.52E-16 |  | *PDGFC* | 0.289 | 3.05E-09 |
| *CHSY1* | 0.389 | 3.72E-16 |  | *OGFOD1* | 0.289 | 3.09E-09 |
| *ZNF281* | 0.389 | 3.86E-16 |  | *RAB18* | 0.289 | 3.11E-09 |
| *MSH6* | 0.389 | 3.89E-16 |  | *ARL4C* | 0.289 | 3.12E-09 |
| *SLC30A4* | 0.389 | 4.04E-16 |  | *SETD3* | 0.289 | 3.17E-09 |
| *SEPTIN11* | 0.389 | 4.17E-16 |  | *ZBTB21* | 0.289 | 3.18E-09 |
| *FBN1* | 0.388 | 5.03E-16 |  | *KPNB1* | 0.288 | 3.27E-09 |
| *GTPBP4* | 0.388 | 5.23E-16 |  | *MEF2D* | 0.288 | 3.27E-09 |
| *MAP4K4* | 0.387 | 5.58E-16 |  | *NT5DC3* | 0.288 | 3.29E-09 |
| *SGO2* | 0.387 | 5.71E-16 |  | *LIPA* | 0.288 | 3.30E-09 |
| *LOX* | 0.387 | 5.80E-16 |  | *AKIRIN1* | 0.288 | 3.32E-09 |
| *LAMP2* | 0.387 | 6.09E-16 |  | *ARHGAP11A* | 0.288 | 3.32E-09 |
| *PRRG1* | 0.387 | 6.10E-16 |  | *KIF1B* | 0.288 | 3.34E-09 |
| *RASSF8* | 0.387 | 6.19E-16 |  | *GPR107* | 0.288 | 3.48E-09 |
| *TLN1* | 0.386 | 7.00E-16 |  | *HTRA2* | 0.288 | 3.52E-09 |
| *CHST11* | 0.386 | 7.73E-16 |  | *SLC39A7* | 0.288 | 3.56E-09 |
| *SEPTIN7* | 0.385 | 8.38E-16 |  | *CTSZ* | 0.287 | 3.65E-09 |
| *B3GNT2* | 0.385 | 8.66E-16 |  | *KLF7* | 0.287 | 3.65E-09 |
| *HIVEP1* | 0.385 | 8.90E-16 |  | *PPP6C* | 0.287 | 3.68E-09 |
| *RAP2C* | 0.385 | 9.13E-16 |  | *DIPK1A* | 0.287 | 3.72E-09 |
| *PLSCR4* | 0.384 | 1.03E-15 |  | *GLIPR1* | 0.287 | 3.73E-09 |
| *UGGT1* | 0.384 | 1.05E-15 |  | *SEC22C* | 0.287 | 3.77E-09 |
| *KPNA2* | 0.382 | 1.43E-15 |  | *FAM210A* | 0.287 | 3.79E-09 |
| *SPTLC1* | 0.382 | 1.52E-15 |  | *MKI67* | 0.287 | 3.79E-09 |
| *HYOU1* | 0.381 | 1.67E-15 |  | *GDI2* | 0.287 | 3.79E-09 |
| *MAP3K20* | 0.381 | 1.82E-15 |  | *ALMS1* | 0.287 | 3.89E-09 |
| *FAM98A* | 0.380 | 2.03E-15 |  | *STIP1* | 0.286 | 4.16E-09 |
| *SLC30A6* | 0.380 | 2.07E-15 |  | *DYNC1LI1* | 0.286 | 4.18E-09 |
| *THBS1* | 0.379 | 2.43E-15 |  | *TTC27* | 0.286 | 4.20E-09 |
| *PLAUR* | 0.379 | 2.43E-15 |  | *UCK2* | 0.286 | 4.21E-09 |
| *CUL2* | 0.379 | 2.44E-15 |  | *PALLD* | 0.286 | 4.22E-09 |
| *TEX2* | 0.379 | 2.64E-15 |  | *NLRP3* | 0.286 | 4.28E-09 |
| *SPTY2D1* | 0.378 | 2.91E-15 |  | *CCN2* | 0.286 | 4.32E-09 |
| *FOXN2* | 0.378 | 3.26E-15 |  | *HIPK1* | 0.286 | 4.34E-09 |
| *ECPAS* | 0.377 | 3.71E-15 |  | *EFR3A* | 0.286 | 4.37E-09 |
| *PICALM* | 0.376 | 4.07E-15 |  | *SMC2* | 0.286 | 4.44E-09 |
| *ADCY3* | 0.376 | 4.15E-15 |  | *AFG3L2* | 0.286 | 4.48E-09 |
| *RRBP1* | 0.376 | 4.30E-15 |  | *SPARC* | 0.286 | 4.58E-09 |
| *SBDS* | 0.375 | 4.94E-15 |  | *ATF1* | 0.286 | 4.59E-09 |
| *SGTB* | 0.375 | 5.20E-15 |  | *TMCC3* | 0.286 | 4.62E-09 |
| *DENND1A* | 0.375 | 5.26E-15 |  | *SMS* | 0.285 | 4.72E-09 |
| *PNMA1* | 0.375 | 5.43E-15 |  | *LRPPRC* | 0.285 | 4.78E-09 |
| *STRN* | 0.374 | 6.43E-15 |  | *NFIX* | 0.285 | 4.84E-09 |
| *MMD* | 0.374 | 6.52E-15 |  | *KCTD10* | 0.285 | 4.92E-09 |
| *KLHL5* | 0.373 | 7.66E-15 |  | *ANXA7* | 0.285 | 4.96E-09 |
| *GSTCD* | 0.372 | 8.48E-15 |  | *TTK* | 0.285 | 5.08E-09 |
| *AQP9* | 0.372 | 8.88E-15 |  | *CUL1* | 0.285 | 5.23E-09 |
| *BPNT2* | 0.372 | 8.97E-15 |  | *RIN2* | 0.285 | 5.24E-09 |
| *MELK* | 0.372 | 8.99E-15 |  | *SPIRE1* | 0.285 | 5.28E-09 |
| *ERLEC1* | 0.371 | 1.04E-14 |  | *RABEP1* | 0.285 | 5.34E-09 |
| *FAM83D* | 0.371 | 1.05E-14 |  | *POSTN* | 0.284 | 5.40E-09 |
| *NUDCD1* | 0.371 | 1.09E-14 |  | *TMCO3* | 0.284 | 5.52E-09 |
| *LTBP2* | 0.370 | 1.18E-14 |  | *USP13* | 0.284 | 5.53E-09 |
| *NFIL3* | 0.370 | 1.20E-14 |  | *PAPSS2* | 0.284 | 5.64E-09 |
| *RPN1* | 0.370 | 1.20E-14 |  | *CDC42BPB* | 0.284 | 5.67E-09 |
| *SEL1L* | 0.370 | 1.25E-14 |  | *RSRC1* | 0.284 | 5.70E-09 |
| *ADAMTS2* | 0.370 | 1.26E-14 |  | *PDCD10* | 0.284 | 5.92E-09 |
| *TBC1D23* | 0.370 | 1.27E-14 |  | *LDLR* | 0.283 | 6.13E-09 |
| *ARNTL2* | 0.370 | 1.31E-14 |  | *NEK2* | 0.283 | 6.15E-09 |
| *BAG2* | 0.370 | 1.32E-14 |  | *FILIP1L* | 0.283 | 6.15E-09 |
| *SHCBP1* | 0.370 | 1.33E-14 |  | *ATP2C1* | 0.283 | 6.26E-09 |
| *TMEM263* | 0.370 | 1.35E-14 |  | *SLC35F6* | 0.283 | 6.36E-09 |
| *GOLGA5* | 0.370 | 1.35E-14 |  | *STAT3* | 0.283 | 6.41E-09 |
| *RIF1* | 0.370 | 1.40E-14 |  | *CHUK* | 0.283 | 6.52E-09 |
| *UGP2* | 0.369 | 1.44E-14 |  | *RIC1* | 0.283 | 6.54E-09 |
| *ROCK1* | 0.369 | 1.45E-14 |  | *CEP112* | 0.283 | 6.63E-09 |
| *DPH3* | 0.369 | 1.52E-14 |  | *ATP2A2* | 0.283 | 6.66E-09 |
| *CEP55* | 0.369 | 1.54E-14 |  | *VCAN* | 0.283 | 6.73E-09 |
| *MSN* | 0.369 | 1.64E-14 |  | *ACAP2* | 0.283 | 6.74E-09 |
| *PLAA* | 0.368 | 1.75E-14 |  | *ST3GAL6* | 0.283 | 6.74E-09 |
| *CENPL* | 0.368 | 1.84E-14 |  | *CD302* | 0.282 | 6.94E-09 |
| *GAPVD1* | 0.368 | 1.94E-14 |  | *KCTD9* | 0.282 | 7.05E-09 |
| *WWC2* | 0.367 | 2.02E-14 |  | *LIX1L* | 0.282 | 7.15E-09 |
| *GPX8* | 0.367 | 2.25E-14 |  | *COL5A3* | 0.282 | 7.32E-09 |
| *KIF20B* | 0.367 | 2.25E-14 |  | *CERS6* | 0.282 | 7.38E-09 |
| *PCNX1* | 0.366 | 2.39E-14 |  | *ARL5A* | 0.282 | 7.41E-09 |
| *PCYOX1* | 0.366 | 2.47E-14 |  | *LRRC8C* | 0.282 | 7.45E-09 |
| *RAB23* | 0.366 | 2.48E-14 |  | *GINS1* | 0.282 | 7.63E-09 |
| *TUBA1C* | 0.366 | 2.68E-14 |  | *MICAL3* | 0.281 | 7.98E-09 |
| *PEA15* | 0.366 | 2.81E-14 |  | *SLC25A24* | 0.281 | 8.02E-09 |
| *GANAB* | 0.365 | 2.93E-14 |  | *TGFA* | 0.281 | 8.12E-09 |
| *CAB39* | 0.365 | 2.96E-14 |  | *KDSR* | 0.281 | 8.17E-09 |
| *DSE* | 0.365 | 3.05E-14 |  | *CDC73* | 0.281 | 8.22E-09 |
| *MAP2K1* | 0.365 | 3.29E-14 |  | *PPAT* | 0.281 | 8.40E-09 |
| *ARHGAP31* | 0.364 | 3.40E-14 |  | *DTD2* | 0.281 | 8.61E-09 |
| *IPO7* | 0.364 | 3.48E-14 |  | *BCAR3* | 0.281 | 8.71E-09 |
| *DCTN1* | 0.364 | 3.70E-14 |  | *RHEB* | 0.281 | 8.74E-09 |
| *WDR43* | 0.364 | 3.72E-14 |  | *FGD6* | 0.281 | 8.76E-09 |
| *EIF2S1* | 0.363 | 4.19E-14 |  | *FAS* | 0.281 | 8.83E-09 |
| *EIF5A2* | 0.363 | 4.26E-14 |  | *RNF169* | 0.281 | 8.85E-09 |
| *YWHAQ* | 0.363 | 4.65E-14 |  | *CDCA8* | 0.281 | 8.89E-09 |
| *MYO5A* | 0.362 | 4.76E-14 |  | *PGM1* | 0.281 | 8.90E-09 |
| *RALB* | 0.362 | 4.86E-14 |  | *PUS7* | 0.280 | 8.95E-09 |
| *IQGAP1* | 0.362 | 5.02E-14 |  | *PBX3* | 0.280 | 9.03E-09 |
| *HEATR1* | 0.362 | 5.05E-14 |  | *STK38L* | 0.280 | 9.12E-09 |
| *CCDC71L* | 0.362 | 5.11E-14 |  | *LSM12* | 0.280 | 9.18E-09 |
| *ADCY7* | 0.362 | 5.19E-14 |  | *SOAT1* | 0.280 | 9.31E-09 |
| *GNA12* | 0.362 | 5.44E-14 |  | *MAP3K3* | 0.280 | 9.43E-09 |
| *SNX6* | 0.362 | 5.55E-14 |  | *FKBP10* | 0.280 | 9.53E-09 |
| *NPC1* | 0.361 | 5.94E-14 |  | *GUCY1B1* | 0.280 | 9.60E-09 |
| *SULF1* | 0.361 | 5.96E-14 |  | *DIXDC1* | 0.280 | 9.61E-09 |
| *AZI2* | 0.361 | 6.24E-14 |  | *TNFAIP3* | 0.280 | 9.70E-09 |
| *COL12A1* | 0.360 | 7.26E-14 |  | *IDH3A* | 0.280 | 9.81E-09 |
| *MTDH* | 0.359 | 7.87E-14 |  | *HTR1D* | 0.280 | 9.82E-09 |
| *CIP2A* | 0.359 | 8.16E-14 |  | *SLC2A13* | 0.280 | 9.99E-09 |
| *TMED10* | 0.359 | 8.66E-14 |  | *DCN* | 0.280 | 1.00E-08 |
| *JCAD* | 0.359 | 9.22E-14 |  | *EIF3J* | 0.280 | 1.01E-08 |
| *ELK3* | 0.358 | 9.31E-14 |  | *MMP8* | 0.279 | 1.01E-08 |
| *CAPZA2* | 0.358 | 9.74E-14 |  | *AKT3* | 0.279 | 1.02E-08 |
| *MCL1* | 0.358 | 9.92E-14 |  | *PRRX1* | 0.279 | 1.02E-08 |
| *TFG* | 0.358 | 1.04E-13 |  | *NOLC1* | 0.279 | 1.02E-08 |
| *TSR1* | 0.358 | 1.07E-13 |  | *MFHAS1* | 0.279 | 1.04E-08 |
| *GREM1* | 0.358 | 1.10E-13 |  | *GBA* | 0.279 | 1.04E-08 |
| *ITGA5* | 0.358 | 1.10E-13 |  | *NAAA* | 0.279 | 1.05E-08 |
| *CKAP5* | 0.357 | 1.16E-13 |  | *HRH1* | 0.279 | 1.06E-08 |
| *MRPL19* | 0.357 | 1.17E-13 |  | *IL1R1* | 0.279 | 1.07E-08 |
| *BUB1* | 0.357 | 1.24E-13 |  | *PXDN* | 0.279 | 1.08E-08 |
| *EIF4G1* | 0.356 | 1.31E-13 |  | *MCM4* | 0.279 | 1.08E-08 |
| *NBAS* | 0.356 | 1.42E-13 |  | *GLMP* | 0.279 | 1.09E-08 |
| *TASOR2* | 0.356 | 1.50E-13 |  | *ADGRA2* | 0.279 | 1.12E-08 |
| *TARS1* | 0.356 | 1.50E-13 |  | *CARM1* | 0.279 | 1.13E-08 |
| *OSTC* | 0.356 | 1.52E-13 |  | *MCEMP1* | 0.279 | 1.14E-08 |
| *MSR1* | 0.355 | 1.62E-13 |  | *CKS1B* | 0.279 | 1.14E-08 |
| *FCGR2A* | 0.355 | 1.72E-13 |  | *PPT1* | 0.278 | 1.17E-08 |
| *MYH9* | 0.355 | 1.73E-13 |  | *ROR2* | 0.278 | 1.20E-08 |
| *PAK2* | 0.355 | 1.79E-13 |  | *COL16A1* | 0.278 | 1.21E-08 |
| *OSBPL8* | 0.354 | 1.85E-13 |  | *SRGN* | 0.278 | 1.22E-08 |
| *MAP7D3* | 0.354 | 1.90E-13 |  | *SERP1* | 0.278 | 1.22E-08 |
| *EMC1* | 0.354 | 1.96E-13 |  | *PITPNB* | 0.278 | 1.23E-08 |
| *YWHAH* | 0.354 | 2.03E-13 |  | *TRMT61B* | 0.278 | 1.27E-08 |
| *MAPKAP1* | 0.354 | 2.13E-13 |  | *COL5A1* | 0.278 | 1.27E-08 |
| *FUBP3* | 0.353 | 2.22E-13 |  | *DCAF6* | 0.278 | 1.28E-08 |
| *DIAPH3* | 0.353 | 2.32E-13 |  | *NUP188* | 0.277 | 1.31E-08 |
| *CEP128* | 0.353 | 2.44E-13 |  | *ATG4C* | 0.277 | 1.31E-08 |
| *PRKDC* | 0.353 | 2.45E-13 |  | *COMMD2* | 0.277 | 1.32E-08 |
| *XPOT* | 0.353 | 2.52E-13 |  | *ALDH18A1* | 0.277 | 1.33E-08 |
| *AURKA* | 0.352 | 2.62E-13 |  | *CCT6A* | 0.277 | 1.33E-08 |
| *ABCC9* | 0.352 | 2.66E-13 |  | *RRAS2* | 0.277 | 1.33E-08 |
| *MCM10* | 0.352 | 2.77E-13 |  | *PROS1* | 0.277 | 1.34E-08 |
| *YKT6* | 0.352 | 2.89E-13 |  | *EIF3A* | 0.277 | 1.34E-08 |
| *INHBA* | 0.351 | 3.21E-13 |  | *KIF1C* | 0.277 | 1.36E-08 |
| *ANAPC1* | 0.351 | 3.26E-13 |  | *ANXA1* | 0.277 | 1.36E-08 |
| *P4HA2* | 0.351 | 3.42E-13 |  | *NAV1* | 0.277 | 1.37E-08 |
| *PSMD1* | 0.351 | 3.45E-13 |  | *SMG8* | 0.277 | 1.37E-08 |
| *FAM126A* | 0.350 | 3.57E-13 |  | *SLC35A2* | 0.277 | 1.39E-08 |
| *BACH1* | 0.350 | 3.81E-13 |  | *C6orf120* | 0.277 | 1.41E-08 |
| *MAPK1* | 0.350 | 4.04E-13 |  | *AKAP12* | 0.277 | 1.42E-08 |
| *CYBRD1* | 0.350 | 4.07E-13 |  | *CMSS1* | 0.277 | 1.42E-08 |
| *WDR3* | 0.350 | 4.14E-13 |  | *ARSJ* | 0.277 | 1.47E-08 |
| *SEC23B* | 0.349 | 4.48E-13 |  | *GMFB* | 0.276 | 1.48E-08 |
| *CMPK1* | 0.349 | 4.63E-13 |  | *SIGLEC7* | 0.276 | 1.49E-08 |
| *SLC4A1AP* | 0.349 | 4.68E-13 |  | *CENPE* | 0.276 | 1.50E-08 |
| *CENPN* | 0.349 | 4.69E-13 |  | *ANXA3* | 0.276 | 1.50E-08 |
| *PDLIM5* | 0.349 | 4.76E-13 |  | *ELOVL7* | 0.276 | 1.51E-08 |
| *TMED5* | 0.348 | 4.99E-13 |  | *DUSP12* | 0.276 | 1.52E-08 |
| *GTF3C4* | 0.348 | 5.01E-13 |  | *CTSB* | 0.276 | 1.54E-08 |
| *RUSC2* | 0.348 | 5.14E-13 |  | *GAS7* | 0.276 | 1.54E-08 |
| *FAM120A* | 0.348 | 5.18E-13 |  | *RCAN1* | 0.276 | 1.55E-08 |
| *COL6A3* | 0.348 | 5.28E-13 |  | *GLIPR2* | 0.276 | 1.55E-08 |
| *MICAL2* | 0.348 | 5.30E-13 |  | *MARS1* | 0.276 | 1.56E-08 |
| *HAUS2* | 0.347 | 5.99E-13 |  | *OSGIN2* | 0.276 | 1.57E-08 |
| *SMC6* | 0.347 | 6.14E-13 |  | *GNB1* | 0.276 | 1.57E-08 |
| *COL15A1* | 0.347 | 6.51E-13 |  | *RAB7A* | 0.276 | 1.57E-08 |
| *PRPF4* | 0.347 | 6.65E-13 |  | *GEM* | 0.276 | 1.58E-08 |
| *UBE2G1* | 0.346 | 7.05E-13 |  | *NUP133* | 0.276 | 1.60E-08 |
| *ATAD2* | 0.346 | 7.13E-13 |  | *PLBD2* | 0.276 | 1.61E-08 |
| *MYO1E* | 0.346 | 7.31E-13 |  | *LCLAT1* | 0.276 | 1.61E-08 |
| *HEG1* | 0.346 | 7.38E-13 |  | *ZC3H14* | 0.276 | 1.61E-08 |
| *COL5A2* | 0.346 | 7.64E-13 |  | *CFL2* | 0.276 | 1.62E-08 |
| *PVR* | 0.346 | 7.65E-13 |  | *LHFPL2* | 0.276 | 1.63E-08 |
| *NRBF2* | 0.346 | 7.78E-13 |  | *HEXB* | 0.276 | 1.63E-08 |
| *PELO* | 0.346 | 7.84E-13 |  | *STAT1* | 0.276 | 1.65E-08 |
| *GMPS* | 0.345 | 8.39E-13 |  | *MATN3* | 0.276 | 1.65E-08 |
| *MFSD14B* | 0.345 | 8.74E-13 |  | *CAP1* | 0.275 | 1.67E-08 |
| *PSMC1* | 0.345 | 9.12E-13 |  | *NIBAN1* | 0.275 | 1.69E-08 |
| *M6PR* | 0.345 | 9.18E-13 |  | *RHOA* | 0.275 | 1.69E-08 |
| *NAA35* | 0.344 | 9.49E-13 |  | *DENND5B* | 0.275 | 1.69E-08 |
| *CANX* | 0.344 | 9.71E-13 |  | *ENPEP* | 0.275 | 1.69E-08 |
| *DUSP3* | 0.344 | 9.79E-13 |  | *FBH1* | 0.275 | 1.73E-08 |
| *HEATR5A* | 0.344 | 1.05E-12 |  | *GOLIM4* | 0.275 | 1.75E-08 |
| *HDGF* | 0.344 | 1.09E-12 |  | *COL1A1* | 0.275 | 1.75E-08 |
| *SOCS5* | 0.343 | 1.12E-12 |  | *EHD2* | 0.275 | 1.76E-08 |
| *SDE2* | 0.343 | 1.19E-12 |  | *TNC* | 0.275 | 1.77E-08 |
| *OSBPL10* | 0.343 | 1.21E-12 |  | *GCC1* | 0.275 | 1.78E-08 |
| *ADAM17* | 0.343 | 1.22E-12 |  | *SH3RF3* | 0.275 | 1.80E-08 |
| *MCMBP* | 0.342 | 1.32E-12 |  | *C1R* | 0.275 | 1.83E-08 |
| *DNAJB9* | 0.342 | 1.35E-12 |  | *ATG9A* | 0.275 | 1.84E-08 |
| *BEND6* | 0.342 | 1.46E-12 |  | *ECM2* | 0.275 | 1.86E-08 |
| *SLC25A43* | 0.342 | 1.48E-12 |  | *REEP3* | 0.274 | 1.92E-08 |
| *TMEM200B* | 0.341 | 1.53E-12 |  | *G2E3* | 0.274 | 1.93E-08 |
| *PDIA4* | 0.341 | 1.54E-12 |  | *P2RY6* | 0.274 | 1.93E-08 |
| *FSTL1* | 0.341 | 1.62E-12 |  | *R3HDM1* | 0.274 | 1.94E-08 |
| *UBXN2A* | 0.341 | 1.68E-12 |  | *PDCL* | 0.274 | 1.95E-08 |
| *PSMD5* | 0.341 | 1.68E-12 |  | *URB2* | 0.274 | 1.97E-08 |
| *GPN1* | 0.340 | 1.82E-12 |  | *TAB3* | 0.274 | 1.99E-08 |
| *ASXL2* | 0.340 | 1.82E-12 |  | *FKBP15* | 0.274 | 2.03E-08 |
| *MSANTD3* | 0.340 | 1.83E-12 |  | *BMP2K* | 0.274 | 2.05E-08 |
| *ACOT9* | 0.340 | 1.85E-12 |  | *PLAGL1* | 0.274 | 2.10E-08 |
| *ARSB* | 0.340 | 1.99E-12 |  | *TMOD3* | 0.274 | 2.10E-08 |
| *YARS1* | 0.340 | 1.99E-12 |  | *EMC2* | 0.273 | 2.13E-08 |
| *ROCK2* | 0.339 | 2.08E-12 |  | *PTPN11* | 0.273 | 2.15E-08 |
| *SYNC* | 0.339 | 2.22E-12 |  | *SGCD* | 0.273 | 2.15E-08 |
| *TGOLN2* | 0.339 | 2.33E-12 |  | *PLA2G7* | 0.273 | 2.15E-08 |
| *CCNA2* | 0.339 | 2.34E-12 |  | *PYGO1* | 0.273 | 2.17E-08 |
| *GART* | 0.339 | 2.37E-12 |  | *SAMHD1* | 0.273 | 2.17E-08 |
| *EIF4H* | 0.339 | 2.41E-12 |  | *RAP2A* | 0.273 | 2.18E-08 |
| *ICAM1* | 0.338 | 2.43E-12 |  | *ATP1A1* | 0.273 | 2.26E-08 |
| *LAMC1* | 0.338 | 2.43E-12 |  | *ARMC1* | 0.273 | 2.29E-08 |
| *PDGFRB* | 0.338 | 2.53E-12 |  | *SFT2D2* | 0.273 | 2.29E-08 |
| *KIRREL1* | 0.338 | 2.55E-12 |  | *S1PR3* | 0.273 | 2.29E-08 |
| *UBE2V2* | 0.338 | 2.56E-12 |  | *ITGAV* | 0.273 | 2.30E-08 |
| *YES1* | 0.338 | 2.60E-12 |  | *KLHL20* | 0.273 | 2.30E-08 |
| *ENAH* | 0.338 | 2.79E-12 |  | *MYH10* | 0.273 | 2.31E-08 |
| *MTHFD1L* | 0.337 | 3.03E-12 |  | *AHNAK* | 0.273 | 2.31E-08 |
| *C11orf24* | 0.337 | 3.04E-12 |  | *SLC11A1* | 0.273 | 2.34E-08 |
| *KPNA6* | 0.337 | 3.19E-12 |  | *ZYX* | 0.273 | 2.37E-08 |
| *NOL10* | 0.337 | 3.32E-12 |  | *TMEM70* | 0.273 | 2.38E-08 |
| *KIF23* | 0.336 | 3.36E-12 |  | *PIK3R4* | 0.273 | 2.38E-08 |
| *NUS1* | 0.336 | 3.37E-12 |  | *SNX9* | 0.273 | 2.39E-08 |
| *PPP1R18* | 0.336 | 3.44E-12 |  | *BZW1* | 0.273 | 2.39E-08 |
| *XPO1* | 0.336 | 3.47E-12 |  | *SLC66A3* | 0.272 | 2.41E-08 |
| *ACTB* | 0.336 | 3.54E-12 |  | *WASHC4* | 0.272 | 2.48E-08 |
| *SH3PXD2B* | 0.336 | 3.56E-12 |  | *GEMIN5* | 0.272 | 2.48E-08 |
| *NCF2* | 0.336 | 3.58E-12 |  | *POLR3G* | 0.272 | 2.49E-08 |
| *GXYLT2* | 0.336 | 3.60E-12 |  | *TAF5L* | 0.272 | 2.50E-08 |
| *SRGAP2B* | 0.336 | 3.68E-12 |  | *IL13RA1* | 0.272 | 2.53E-08 |
| *ACTR3* | 0.336 | 3.75E-12 |  | *GATA6* | 0.272 | 2.54E-08 |
| *CHCHD3* | 0.335 | 3.94E-12 |  | *CIZ1* | 0.272 | 2.56E-08 |
| *CLMP* | 0.335 | 4.02E-12 |  | *OSBPL11* | 0.272 | 2.57E-08 |
| *ADAM12* | 0.335 | 4.06E-12 |  | *MPP1* | 0.272 | 2.63E-08 |
| *TMEM65* | 0.335 | 4.26E-12 |  | *TGFBRAP1* | 0.272 | 2.65E-08 |
| *NIPA2* | 0.335 | 4.35E-12 |  | *RTKN* | 0.272 | 2.67E-08 |
| *STIL* | 0.335 | 4.37E-12 |  | *FAM20C* | 0.272 | 2.71E-08 |
| *FCF1* | 0.335 | 4.38E-12 |  | *LRRK2* | 0.271 | 2.75E-08 |
| *CNN3* | 0.335 | 4.48E-12 |  | *ZNF106* | 0.271 | 2.77E-08 |
| *LOXL2* | 0.335 | 4.50E-12 |  | *DOCK4* | 0.271 | 2.80E-08 |
| *UBAP1* | 0.334 | 4.58E-12 |  | *PPP3CA* | 0.271 | 2.84E-08 |
| *F2R* | 0.334 | 4.68E-12 |  | *CLSPN* | 0.271 | 2.91E-08 |
| *CPSF2* | 0.334 | 4.84E-12 |  | *ZNF770* | 0.271 | 2.92E-08 |
| *GNG12* | 0.334 | 4.85E-12 |  | *NCAPG2* | 0.271 | 2.94E-08 |
| *DCAF10* | 0.334 | 4.86E-12 |  | *TXNDC9* | 0.271 | 3.02E-08 |
| *MAP4* | 0.334 | 5.02E-12 |  | *UTP20* | 0.271 | 3.03E-08 |
| *HSPG2* | 0.334 | 5.27E-12 |  | *KIF11* | 0.271 | 3.03E-08 |
| *TEAD1* | 0.333 | 5.32E-12 |  | *CCNY* | 0.271 | 3.07E-08 |
| *SLC16A1* | 0.333 | 5.32E-12 |  | *CD274* | 0.270 | 3.16E-08 |
| *CHSY3* | 0.333 | 5.37E-12 |  | *PRPS1* | 0.270 | 3.20E-08 |
| *RFK* | 0.333 | 5.40E-12 |  | *DNAJC3* | 0.270 | 3.22E-08 |
| *PRDX4* | 0.333 | 5.41E-12 |  | *USP39* | 0.270 | 3.26E-08 |
| *KLF6* | 0.333 | 5.69E-12 |  | *USP1* | 0.270 | 3.33E-08 |
| *GPATCH4* | 0.333 | 5.76E-12 |  | *EMP1* | 0.270 | 3.33E-08 |
| *NUP205* | 0.333 | 5.78E-12 |  | *RBM12* | 0.270 | 3.37E-08 |
| *NUP160* | 0.332 | 6.20E-12 |  | *HUWE1* | 0.270 | 3.40E-08 |
| *TRAM2* | 0.332 | 6.34E-12 |  | *STK17B* | 0.270 | 3.40E-08 |
| *COPA* | 0.332 | 6.42E-12 |  | *CYRIB* | 0.270 | 3.42E-08 |
| *GARS1* | 0.332 | 6.46E-12 |  | *TAF4B* | 0.270 | 3.43E-08 |
| *GPC6* | 0.332 | 6.51E-12 |  | *GLG1* | 0.269 | 3.49E-08 |
| *POGLUT3* | 0.332 | 6.70E-12 |  | *B3GNT10* | 0.269 | 3.53E-08 |
| *GNAI3* | 0.332 | 7.07E-12 |  | *SMG6* | 0.269 | 3.64E-08 |
| *LYSMD3* | 0.331 | 7.26E-12 |  | *F3* | 0.269 | 3.65E-08 |
| *TEX10* | 0.331 | 7.33E-12 |  | *SFRP2* | 0.269 | 3.79E-08 |
| *ENOX2* | 0.331 | 7.33E-12 |  | *MEF2A* | 0.269 | 3.86E-08 |
| *RAB27A* | 0.331 | 7.45E-12 |  | *PRKD3* | 0.269 | 3.87E-08 |
| *BICC1* | 0.331 | 7.46E-12 |  | *SCFD1* | 0.269 | 3.89E-08 |
| *FBXO11* | 0.331 | 7.54E-12 |  | *COA7* | 0.269 | 3.91E-08 |
| *NAA15* | 0.331 | 7.78E-12 |  | *CALR* | 0.268 | 3.93E-08 |
| *ITPRIP* | 0.331 | 7.93E-12 |  | *FANCB* | 0.268 | 3.94E-08 |
| *LTBP1* | 0.331 | 8.24E-12 |  | *PYCR1* | 0.268 | 4.02E-08 |
| *ADAMTS4* | 0.330 | 8.43E-12 |  | *PPIL1* | 0.268 | 4.06E-08 |
| *ABL1* | 0.330 | 8.48E-12 |  | *SIGMAR1* | 0.268 | 4.08E-08 |
| *DYRK3* | 0.330 | 9.23E-12 |  | *AXL* | 0.268 | 4.14E-08 |
| *CENPI* | 0.330 | 9.28E-12 |  | *NRDC* | 0.268 | 4.16E-08 |
| *FMNL2* | 0.330 | 9.32E-12 |  | *COLEC12* | 0.268 | 4.18E-08 |
| *FN1* | 0.330 | 9.37E-12 |  | *KCTD5* | 0.268 | 4.27E-08 |
| *SOD2* | 0.330 | 9.46E-12 |  | *VAPA* | 0.268 | 4.29E-08 |
| *NUP155* | 0.330 | 9.73E-12 |  | *CTPS1* | 0.268 | 4.29E-08 |
| *CERCAM* | 0.329 | 1.03E-11 |  | *ANTXR2* | 0.268 | 4.36E-08 |
| *COG5* | 0.329 | 1.03E-11 |  | *C1D* | 0.267 | 4.45E-08 |
| *TDO2* | 0.329 | 1.06E-11 |  | *UFSP2* | 0.267 | 4.48E-08 |
| *LRRFIP2* | 0.329 | 1.06E-11 |  | *PAPPA* | 0.267 | 4.50E-08 |
| *CRISPLD2* | 0.329 | 1.07E-11 |  | *TTC7A* | 0.267 | 4.56E-08 |
| *SRBD1* | 0.329 | 1.07E-11 |  | *ADAM9* | 0.267 | 4.57E-08 |
| *PLOD1* | 0.329 | 1.08E-11 |  | *ITPR3* | 0.267 | 4.57E-08 |
| *TUBB2A* | 0.329 | 1.09E-11 |  | *CEP170* | 0.267 | 4.62E-08 |
| *CHM* | 0.329 | 1.13E-11 |  | *MTMR2* | 0.267 | 4.66E-08 |
| *ADAR* | 0.328 | 1.18E-11 |  | *ITPR2* | 0.267 | 4.66E-08 |
| *NRP1* | 0.328 | 1.19E-11 |  | *PTBP3* | 0.267 | 4.67E-08 |
| *ZDHHC9* | 0.328 | 1.19E-11 |  | *DHX9* | 0.267 | 4.77E-08 |
| *TMED7* | 0.328 | 1.19E-11 |  | *MED14* | 0.267 | 4.80E-08 |
| *NCBP1* | 0.328 | 1.21E-11 |  | *ZMPSTE24* | 0.267 | 4.86E-08 |
| *VAMP7* | 0.328 | 1.25E-11 |  | *COPS8* | 0.267 | 4.87E-08 |
| *CAVIN1* | 0.328 | 1.25E-11 |  | *MPDZ* | 0.267 | 4.94E-08 |
| *RECK* | 0.328 | 1.26E-11 |  | *FAM124A* | 0.267 | 4.95E-08 |
| *SACS* | 0.328 | 1.28E-11 |  | *CDCP1* | 0.267 | 4.97E-08 |
| *CTHRC1* | 0.328 | 1.32E-11 |  | *XPO5* | 0.267 | 4.97E-08 |
| *LAMA4* | 0.327 | 1.50E-11 |  | *LUM* | 0.266 | 5.04E-08 |
| *C5AR1* | 0.327 | 1.53E-11 |  | *NT5E* | 0.266 | 5.08E-08 |
| *RAD18* | 0.327 | 1.54E-11 |  | *CHST15* | 0.266 | 5.08E-08 |
| *ZBTB38* | 0.326 | 1.58E-11 |  | *COL6A2* | 0.266 | 5.12E-08 |
| *AIMP2* | 0.326 | 1.61E-11 |  | *BUB1B* | 0.266 | 5.17E-08 |
| *DESI2* | 0.326 | 1.61E-11 |  | *RAPGEF1* | 0.266 | 5.27E-08 |
| *YIPF5* | 0.326 | 1.65E-11 |  | *TRPC1* | 0.266 | 5.31E-08 |
| *PPP2R5E* | 0.326 | 1.65E-11 |  | *LRRC40* | 0.266 | 5.32E-08 |
| *AGPS* | 0.326 | 1.66E-11 |  | *STOM* | 0.266 | 5.35E-08 |
| *ALDH1B1* | 0.326 | 1.66E-11 |  | *P2RX7* | 0.266 | 5.37E-08 |
| *MAGT1* | 0.326 | 1.76E-11 |  | *SLC44A1* | 0.266 | 5.37E-08 |
| *STX2* | 0.325 | 1.88E-11 |  | *EPS15* | 0.266 | 5.45E-08 |
| *PAFAH1B1* | 0.325 | 1.92E-11 |  | *HSP90B1* | 0.266 | 5.56E-08 |
| *FNDC3B* | 0.325 | 1.96E-11 |  | *BMP1* | 0.266 | 5.58E-08 |
| *CCT4* | 0.325 | 1.98E-11 |  | *IL7R* | 0.265 | 5.65E-08 |
| *ATP13A3* | 0.325 | 1.98E-11 |  | *MRPL3* | 0.265 | 5.70E-08 |
| *USP32* | 0.325 | 2.07E-11 |  | *API5* | 0.265 | 5.74E-08 |
| *SERPINB9* | 0.324 | 2.12E-11 |  | *CRY1* | 0.265 | 5.77E-08 |
| *ANTXR1* | 0.324 | 2.21E-11 |  | *TMEM214* | 0.265 | 5.78E-08 |
| *QKI* | 0.324 | 2.33E-11 |  | *SEH1L* | 0.265 | 5.92E-08 |
| *EPB41L2* | 0.324 | 2.40E-11 |  | *UHRF1BP1* | 0.265 | 5.97E-08 |
| *INCENP* | 0.323 | 2.53E-11 |  | *DSG2* | 0.265 | 6.01E-08 |
| *RAP1A* | 0.323 | 2.58E-11 |  | *KIF5B* | 0.265 | 6.06E-08 |
| *PIK3CA* | 0.323 | 2.61E-11 |  | *FNDC1* | 0.265 | 6.08E-08 |
| *CERS2* | 0.323 | 2.62E-11 |  | *UBE2A* | 0.265 | 6.09E-08 |
| *ASCC3* | 0.323 | 2.67E-11 |  | *MTMR6* | 0.265 | 6.14E-08 |
| *VIRMA* | 0.323 | 2.68E-11 |  | *NANP* | 0.265 | 6.22E-08 |
| *EDNRA* | 0.323 | 2.76E-11 |  | *TM6SF1* | 0.265 | 6.30E-08 |
| *WDR44* | 0.322 | 2.80E-11 |  | *IL18R1* | 0.264 | 6.32E-08 |
| *SPTBN1* | 0.322 | 2.82E-11 |  | *MIB1* | 0.264 | 6.36E-08 |
| *FOXM1* | 0.322 | 2.94E-11 |  | *VASN* | 0.264 | 6.48E-08 |
| *SDCBP* | 0.322 | 2.98E-11 |  | *MAMLD1* | 0.264 | 6.54E-08 |
| *KLF10* | 0.322 | 3.00E-11 |  | *UBE2E2* | 0.264 | 6.55E-08 |
| *RRP1B* | 0.322 | 3.02E-11 |  | *AFAP1L1* | 0.264 | 6.61E-08 |
| *NUP153* | 0.322 | 3.03E-11 |  | *SERPINE1* | 0.264 | 6.75E-08 |
| *FKBP14* | 0.322 | 3.11E-11 |  | *FBXO32* | 0.264 | 6.80E-08 |
| *IPO11* | 0.322 | 3.12E-11 |  | *NOMO3* | 0.264 | 6.84E-08 |
| *BNC2* | 0.322 | 3.13E-11 |  | *CCR8* | 0.264 | 6.99E-08 |
| *IKBIP* | 0.322 | 3.17E-11 |  | *CLEC5A* | 0.264 | 7.00E-08 |
| *VCP* | 0.321 | 3.32E-11 |  | *DNAJC10* | 0.263 | 7.11E-08 |
| *MTPN* | 0.321 | 3.36E-11 |  | *IRAK1* | 0.263 | 7.13E-08 |
| *RPS6KA3* | 0.321 | 3.40E-11 |  | *PIK3CB* | 0.263 | 7.14E-08 |
| *GLT8D2* | 0.321 | 3.43E-11 |  | *PARVB* | 0.263 | 7.17E-08 |
| *ERGIC2* | 0.321 | 3.60E-11 |  | *GDAP2* | 0.263 | 7.17E-08 |
| *NUCB2* | 0.321 | 3.74E-11 |  | *PDZD8* | 0.263 | 7.22E-08 |
| *PRIM2* | 0.321 | 3.75E-11 |  | *ITGB5* | 0.263 | 7.22E-08 |
| *DLGAP5* | 0.320 | 4.00E-11 |  | *NDEL1* | 0.263 | 7.37E-08 |
| *ARL13B* | 0.320 | 4.18E-11 |  | *KPNA3* | 0.263 | 7.47E-08 |
| *ADGRE2* | 0.319 | 4.38E-11 |  | *MFSD14A* | 0.263 | 7.54E-08 |
| *SNX10* | 0.319 | 4.39E-11 |  | *PPP2R5D* | 0.263 | 7.77E-08 |
| *PCDHGA12* | 0.319 | 4.40E-11 |  | *TOR1B* | 0.263 | 7.89E-08 |
| *PPFIBP1* | 0.319 | 4.46E-11 |  | *DOCK11* | 0.263 | 7.90E-08 |
| *NRBP1* | 0.319 | 4.89E-11 |  | *PCDHGA4* | 0.263 | 7.97E-08 |
| *GPR176* | 0.319 | 5.02E-11 |  | *TMCC1* | 0.262 | 8.06E-08 |
| *DNAJB4* | 0.318 | 5.07E-11 |  | *ANKIB1* | 0.262 | 8.10E-08 |
| *IPPK* | 0.318 | 5.09E-11 |  | *HAVCR2* | 0.262 | 8.11E-08 |
| *STAG1* | 0.318 | 5.70E-11 |  | *KLHL7* | 0.262 | 8.32E-08 |
| *CSE1L* | 0.318 | 5.75E-11 |  | *BRCA1* | 0.262 | 8.38E-08 |
| *SRP54* | 0.317 | 5.94E-11 |  | *STAM* | 0.262 | 8.45E-08 |
| *BCORL1* | 0.317 | 6.06E-11 |  | *STARD13* | 0.262 | 8.66E-08 |
| *KDM1B* | 0.317 | 6.50E-11 |  | *OSM* | 0.262 | 8.68E-08 |
| *PUM3* | 0.317 | 6.53E-11 |  | *SNX30* | 0.262 | 8.69E-08 |
| *MFAP3* | 0.316 | 6.92E-11 |  | *CDKN3* | 0.262 | 8.69E-08 |
| *ERC1* | 0.316 | 6.96E-11 |  | *SP3* | 0.262 | 8.72E-08 |
| *C1S* | 0.316 | 7.14E-11 |  | *NCL* | 0.262 | 8.75E-08 |
| *RLIM* | 0.316 | 7.24E-11 |  | *EIF2S3* | 0.262 | 8.84E-08 |
| *AGFG1* | 0.316 | 7.35E-11 |  | *VEGFC* | 0.262 | 8.87E-08 |
| *ITGBL1* | 0.316 | 7.36E-11 |  | *FNDC4* | 0.262 | 8.87E-08 |
| *ATL2* | 0.316 | 7.41E-11 |  | *PPP1CB* | 0.261 | 9.09E-08 |
| *APBB2* | 0.316 | 7.46E-11 |  | *DNAJC13* | 0.261 | 9.14E-08 |
| *KIF4A* | 0.316 | 7.47E-11 |  | *PTGDR* | 0.261 | 9.15E-08 |
| *TPM4* | 0.316 | 7.62E-11 |  | *HELB* | 0.261 | 9.28E-08 |
| *DERL1* | 0.315 | 7.84E-11 |  | *ITSN1* | 0.261 | 9.28E-08 |
| *NRP2* | 0.315 | 8.24E-11 |  | *GOLGA4* | 0.261 | 9.30E-08 |
| *MACF1* | 0.315 | 8.73E-11 |  | *ADAM19* | 0.261 | 9.41E-08 |
| *ARFGAP3* | 0.315 | 8.97E-11 |  | *GOSR2* | 0.261 | 9.41E-08 |
| *TCP11L1* | 0.314 | 9.40E-11 |  | *LRRC42* | 0.261 | 9.42E-08 |
| *CYTH3* | 0.314 | 9.51E-11 |  | *TUT7* | 0.261 | 9.52E-08 |
| *MOB1A* | 0.314 | 9.62E-11 |  | *EPN2* | 0.261 | 9.60E-08 |
| *CALM2* | 0.314 | 9.63E-11 |  | *ITGA1* | 0.261 | 9.73E-08 |
| *P4HA3* | 0.314 | 9.84E-11 |  | *ARMT1* | 0.261 | 9.74E-08 |
| *OLFML2B* | 0.314 | 1.03E-10 |  | *RGP1* | 0.261 | 9.80E-08 |
| *PANX1* | 0.314 | 1.03E-10 |  | *NFE2L1* | 0.261 | 9.82E-08 |
| *LIMK1* | 0.313 | 1.05E-10 |  | *CDK12* | 0.261 | 9.96E-08 |
| *MTAP* | 0.313 | 1.06E-10 |  | *SSR1* | 0.261 | 1.01E-07 |
| *DENND5A* | 0.313 | 1.13E-10 |  | *BAZ1B* | 0.260 | 1.03E-07 |
| *AUNIP* | 0.313 | 1.14E-10 |  | *SSRP1* | 0.260 | 1.04E-07 |
| *CLTC* | 0.313 | 1.15E-10 |  | *PDS5B* | 0.260 | 1.04E-07 |
| *ADAMTS12* | 0.313 | 1.15E-10 |  | *SEC14L1* | 0.260 | 1.04E-07 |
| *SLC25A51* | 0.313 | 1.18E-10 |  | *BICD1* | 0.260 | 1.05E-07 |
| *KLF11* | 0.313 | 1.18E-10 |  | *SELENOI* | 0.260 | 1.05E-07 |
| *KCMF1* | 0.312 | 1.22E-10 |  | *SLC39A10* | 0.260 | 1.07E-07 |
| *CALD1* | 0.312 | 1.23E-10 |  | *GNAQ* | 0.260 | 1.08E-07 |
| *WWTR1* | 0.312 | 1.24E-10 |  | *PNP* | 0.260 | 1.09E-07 |
| *SUSD6* | 0.312 | 1.26E-10 |  | *EIF1AX* | 0.260 | 1.09E-07 |
| *SERBP1* | 0.312 | 1.30E-10 |  | *MAN1A1* | 0.260 | 1.11E-07 |
| *USP9X* | 0.312 | 1.31E-10 |  | *PGM2L1* | 0.260 | 1.13E-07 |
| *DNAJA1* | 0.311 | 1.38E-10 |  | *HABP4* | 0.260 | 1.13E-07 |
| *AHCTF1* | 0.311 | 1.39E-10 |  | *EVC* | 0.259 | 1.13E-07 |
| *FERMT2* | 0.311 | 1.40E-10 |  | *SLAMF8* | 0.259 | 1.16E-07 |
| *ARHGAP29* | 0.311 | 1.41E-10 |  | *EXOSC3* | 0.259 | 1.18E-07 |
| *TMED2* | 0.311 | 1.43E-10 |  | *HNRNPK* | 0.259 | 1.19E-07 |
| *NMT2* | 0.311 | 1.45E-10 |  | *P3H1* | 0.259 | 1.20E-07 |
| *GFM1* | 0.311 | 1.46E-10 |  | *SLC39A8* | 0.259 | 1.20E-07 |
| *CGAS* | 0.311 | 1.48E-10 |  | *OAT* | 0.259 | 1.21E-07 |
| *ZFP91* | 0.311 | 1.58E-10 |  | *ZNF143* | 0.259 | 1.22E-07 |
| *SH2B3* | 0.311 | 1.59E-10 |  | *KLF9* | 0.259 | 1.22E-07 |
| *HASPIN* | 0.310 | 1.61E-10 |  | *DOLK* | 0.259 | 1.25E-07 |
| *SIRPA* | 0.310 | 1.62E-10 |  | *OLFML1* | 0.259 | 1.25E-07 |
| *C1GALT1* | 0.310 | 1.67E-10 |  | *DBF4* | 0.259 | 1.25E-07 |
| *TTC26* | 0.310 | 1.68E-10 |  | *KCNE1* | 0.258 | 1.28E-07 |
| *WIPI1* | 0.310 | 1.69E-10 |  | *POLR1G* | 0.258 | 1.30E-07 |
| *IARS2* | 0.310 | 1.73E-10 |  | *STAG2* | 0.258 | 1.31E-07 |
| *GALNT10* | 0.310 | 1.76E-10 |  | *CTNNB1* | 0.258 | 1.31E-07 |
| *MCM6* | 0.310 | 1.77E-10 |  | *SNX8* | 0.258 | 1.32E-07 |
| *ITPRID2* | 0.310 | 1.77E-10 |  | *PRDX6* | 0.258 | 1.33E-07 |
| *ADAMTS3* | 0.310 | 1.78E-10 |  | *SLFN11* | 0.258 | 1.36E-07 |
| *CDC27* | 0.309 | 1.86E-10 |  | *RTCA* | 0.258 | 1.36E-07 |
| *TMEM38B* | 0.309 | 1.86E-10 |  | *DNAAF5* | 0.258 | 1.37E-07 |
| *PLXNC1* | 0.309 | 1.96E-10 |  | *PSMC2* | 0.258 | 1.41E-07 |
| *MAPK14* | 0.309 | 1.96E-10 |  | *PDGFRL* | 0.258 | 1.41E-07 |
| *SPTAN1* | 0.309 | 2.05E-10 |  | *PLK1* | 0.258 | 1.42E-07 |
| *OSBPL1A* | 0.309 | 2.05E-10 |  | *EDEM3* | 0.258 | 1.42E-07 |
| *ORC1* | 0.309 | 2.07E-10 |  | *SLC17A9* | 0.257 | 1.43E-07 |
| *PJA2* | 0.309 | 2.10E-10 |  | *RFC1* | 0.257 | 1.44E-07 |
| *FAM91A1* | 0.309 | 2.11E-10 |  | *LMCD1* | 0.257 | 1.45E-07 |
| *SETX* | 0.308 | 2.12E-10 |  | *MTMR12* | 0.257 | 1.46E-07 |
| *UACA* | 0.308 | 2.13E-10 |  | *MMS22L* | 0.257 | 1.46E-07 |
| *EXOC5* | 0.308 | 2.14E-10 |  | *REXO2* | 0.257 | 1.48E-07 |
| *CTSL* | 0.308 | 2.15E-10 |  | *SRPX* | 0.257 | 1.48E-07 |
| *USP34* | 0.308 | 2.15E-10 |  | *SEC13* | 0.257 | 1.49E-07 |
| *CCIN* | 0.308 | 2.16E-10 |  | *RIPK2* | 0.257 | 1.51E-07 |
| *LRRC58* | 0.308 | 2.18E-10 |  | *SUMF2* | 0.257 | 1.51E-07 |
| *GOLM1* | 0.308 | 2.25E-10 |  | *CNTLN* | 0.257 | 1.59E-07 |
| *HACD2* | 0.308 | 2.30E-10 |  | *HBP1* | 0.257 | 1.60E-07 |
| *AMOTL2* | 0.308 | 2.36E-10 |  | *ASPM* | 0.257 | 1.60E-07 |
| *ETF1* | 0.308 | 2.39E-10 |  | *APOOL* | 0.256 | 1.61E-07 |
| *CCNYL1* | 0.308 | 2.41E-10 |  | *GOLPH3* | 0.256 | 1.61E-07 |
| *COL4A1* | 0.308 | 2.44E-10 |  | *B4GALT5* | 0.256 | 1.63E-07 |
| *FKTN* | 0.307 | 2.50E-10 |  | *KNSTRN* | 0.256 | 1.64E-07 |
| *COL3A1* | 0.307 | 2.50E-10 |  | *SGO1* | 0.256 | 1.64E-07 |
| *LYN* | 0.307 | 2.50E-10 |  | *AP2A1* | 0.256 | 1.64E-07 |
| *C1orf112* | 0.307 | 2.56E-10 |  | *NAA50* | 0.256 | 1.65E-07 |
| *PDE4DIP* | 0.307 | 2.66E-10 |  | *CCNB1* | 0.256 | 1.66E-07 |
| *SCRN1* | 0.307 | 2.69E-10 |  | *PGK1* | 0.256 | 1.66E-07 |
| *TIMP2* | 0.307 | 2.74E-10 |  | *YY1AP1* | 0.256 | 1.75E-07 |
| *TRAPPC10* | 0.307 | 2.75E-10 |  | *BHLHA15* | 0.256 | 1.77E-07 |
| *SMC4* | 0.307 | 2.77E-10 |  | *TIPRL* | 0.256 | 1.77E-07 |
| *DUSP14* | 0.306 | 2.88E-10 |  | *EPHB2* | 0.256 | 1.77E-07 |
| *CARNMT1* | 0.306 | 2.90E-10 |  | *TPP1* | 0.256 | 1.79E-07 |
| *PRDM1* | 0.306 | 3.01E-10 |  | *PAM* | 0.255 | 1.81E-07 |
| *DYSF* | 0.306 | 3.01E-10 |  | *ARHGEF17* | 0.255 | 1.83E-07 |
| *CAD* | 0.306 | 3.05E-10 |  | *SAR1A* | 0.255 | 1.84E-07 |
| *MYADM* | 0.306 | 3.06E-10 |  | *CLIP1* | 0.255 | 1.87E-07 |
| *IGF2BP2* | 0.306 | 3.18E-10 |  | *PRMT5* | 0.255 | 1.89E-07 |
| *CAV2* | 0.305 | 3.22E-10 |  | *RPS6KC1* | 0.255 | 1.89E-07 |
| *PARVA* | 0.305 | 3.24E-10 |  | *SPIN1* | 0.255 | 1.91E-07 |
| *FMO1* | 0.305 | 3.27E-10 |  | *TDRD7* | 0.255 | 1.93E-07 |
| *EVI5* | 0.305 | 3.32E-10 |  | *KLHL15* | 0.255 | 1.99E-07 |
| *PEAK1* | 0.305 | 3.51E-10 |  | *SLC2A3* | 0.255 | 1.99E-07 |
| *ARF6* | 0.305 | 3.51E-10 |  | *SMCO4* | 0.255 | 1.99E-07 |
| *PPP2R3A* | 0.305 | 3.51E-10 |  | *MPP6* | 0.254 | 2.02E-07 |
| *CDH11* | 0.305 | 3.55E-10 |  | *NAA30* | 0.254 | 2.02E-07 |
| *SUV39H2* | 0.305 | 3.55E-10 |  | *TNFRSF12A* | 0.254 | 2.03E-07 |
| *KATNAL1* | 0.305 | 3.58E-10 |  | *L2HGDH* | 0.254 | 2.04E-07 |
| *FGD1* | 0.305 | 3.60E-10 |  | *CASS4* | 0.254 | 2.05E-07 |
| *SLC6A9* | 0.305 | 3.66E-10 |  | *ZFP92* | 0.254 | 2.06E-07 |
| *DEPDC1* | 0.305 | 3.70E-10 |  | *TRAPPC6B* | 0.254 | 2.06E-07 |
| *BCL9L* | 0.304 | 3.75E-10 |  | *ARMC9* | 0.254 | 2.08E-07 |
| *ADCY9* | 0.304 | 3.80E-10 |  | *SMIM3* | 0.254 | 2.09E-07 |
| *SET* | 0.304 | 3.87E-10 |  | *GAS1* | 0.254 | 2.09E-07 |
| *JOSD1* | 0.304 | 3.95E-10 |  | *WBP11* | 0.254 | 2.11E-07 |
| *FLVCR2* | 0.304 | 3.99E-10 |  | *TPBG* | 0.254 | 2.11E-07 |
| *STARD3NL* | 0.304 | 4.08E-10 |  | *ANGPTL2* | 0.254 | 2.12E-07 |
| *WDHD1* | 0.304 | 4.13E-10 |  | *CENPF* | 0.254 | 2.13E-07 |
| *PRKAR2A* | 0.304 | 4.19E-10 |  | *IAH1* | 0.254 | 2.14E-07 |
| *UHMK1* | 0.304 | 4.25E-10 |  | *KIF24* | 0.254 | 2.14E-07 |
| *HSPD1* | 0.303 | 4.32E-10 |  | *EXT1* | 0.254 | 2.19E-07 |
| *PATL1* | 0.303 | 4.33E-10 |  | *MCUR1* | 0.254 | 2.22E-07 |
| *NLN* | 0.303 | 4.37E-10 |  | *PHLPP2* | 0.254 | 2.22E-07 |
| *MRAS* | 0.303 | 4.45E-10 |  | *CHEK1* | 0.254 | 2.22E-07 |
| *PDE4B* | 0.303 | 4.53E-10 |  | *GLIS3* | 0.254 | 2.23E-07 |
| *MYO1B* | 0.303 | 4.59E-10 |  | *OGDH* | 0.254 | 2.24E-07 |
| *ABCB10* | 0.303 | 4.65E-10 |  | *RHBDF2* | 0.253 | 2.28E-07 |
| *CDK14* | 0.303 | 4.65E-10 |  | *SLMAP* | 0.253 | 2.29E-07 |
| *IMMT* | 0.303 | 4.68E-10 |  | *SVEP1* | 0.253 | 2.29E-07 |
| *FER* | 0.303 | 4.77E-10 |  | *PODXL* | 0.253 | 2.30E-07 |
| *FPR2* | 0.302 | 4.93E-10 |  | *ATP11A* | 0.253 | 2.31E-07 |
| *VCL* | 0.302 | 4.93E-10 |  | *DNAJC9* | 0.253 | 2.32E-07 |
| *RACGAP1* | 0.302 | 4.98E-10 |  | *IL27RA* | 0.253 | 2.33E-07 |
| *C9orf64* | 0.302 | 5.09E-10 |  | *SAP130* | 0.253 | 2.33E-07 |
| *SGIP1* | 0.302 | 5.21E-10 |  | *TNPO3* | 0.253 | 2.35E-07 |
| *GPR180* | 0.302 | 5.37E-10 |  | *CD163* | 0.253 | 2.38E-07 |
| *MOB3B* | 0.302 | 5.38E-10 |  | *CD93* | 0.253 | 2.39E-07 |
| *COPG1* | 0.302 | 5.42E-10 |  | *TBC1D8B* | 0.253 | 2.40E-07 |
| *POGLUT2* | 0.302 | 5.49E-10 |  | *BBX* | 0.253 | 2.41E-07 |
| *SLC30A7* | 0.302 | 5.55E-10 |  | *AEBP1* | 0.253 | 2.41E-07 |
| *NR3C1* | 0.302 | 5.59E-10 |  | *LDLRAD3* | 0.253 | 2.42E-07 |
| *KIF18A* | 0.301 | 5.62E-10 |  | *ERCC6L* | 0.253 | 2.43E-07 |
| *FEZ2* | 0.301 | 5.63E-10 |  | *NCK1* | 0.253 | 2.45E-07 |
| *TRIO* | 0.301 | 5.67E-10 |  | *SPIN4* | 0.253 | 2.46E-07 |
| *HAT1* | 0.301 | 5.68E-10 |  | *FMN1* | 0.253 | 2.50E-07 |
| *PPP4R3B* | 0.301 | 5.78E-10 |  | *NAB1* | 0.253 | 2.51E-07 |
| *SLC31A2* | 0.301 | 5.80E-10 |  | *NUP98* | 0.253 | 2.52E-07 |
| *TMEM39A* | 0.301 | 5.81E-10 |  | *FANCM* | 0.253 | 2.52E-07 |
| *NTAQ1* | 0.301 | 5.90E-10 |  | *ANP32E* | 0.252 | 2.54E-07 |
| *WNK1* | 0.301 | 6.17E-10 |  | *ANXA6* | 0.252 | 2.55E-07 |
| *SYDE1* | 0.301 | 6.22E-10 |  | *URB1* | 0.252 | 2.56E-07 |
| *EIF2AK2* | 0.301 | 6.26E-10 |  | *SLC41A2* | 0.252 | 2.67E-07 |
| *DDX1* | 0.301 | 6.42E-10 |  | *EPB41L5* | 0.252 | 2.69E-07 |
| *NCAPG* | 0.301 | 6.42E-10 |  | *CCT5* | 0.252 | 2.71E-07 |
| *SUMO3* | 0.300 | 6.59E-10 |  | *MSRB3* | 0.252 | 2.72E-07 |
| *CPD* | 0.300 | 6.65E-10 |  | *GALK2* | 0.252 | 2.74E-07 |
| *RNF216* | 0.300 | 6.90E-10 |  | *CCZ1B* | 0.252 | 2.77E-07 |
| *CHST3* | 0.300 | 6.96E-10 |  | *LIF* | 0.252 | 2.78E-07 |
| *ADIPOR2* | 0.300 | 7.00E-10 |  | *EIF2S3B* | 0.252 | 2.79E-07 |
| *TAB2* | 0.300 | 7.00E-10 |  | *MYC* | 0.252 | 2.79E-07 |
| *COL1A2* | 0.300 | 7.21E-10 |  | *RPA1* | 0.252 | 2.80E-07 |
| *SPHK1* | 0.299 | 7.38E-10 |  | *BARD1* | 0.252 | 2.80E-07 |
| *P4HB* | 0.299 | 7.38E-10 |  | *H2BC12* | 0.251 | 2.84E-07 |
| *RNF26* | 0.299 | 7.72E-10 |  | *NIN* | 0.251 | 2.85E-07 |
| *COL4A2* | 0.299 | 7.77E-10 |  | *IPO5* | 0.251 | 2.87E-07 |
| *TPST1* | 0.299 | 7.90E-10 |  | *OXCT1* | 0.251 | 2.87E-07 |
| *FGD4* | 0.299 | 7.95E-10 |  | *ACOT7* | 0.251 | 2.89E-07 |
| *RC3H2* | 0.299 | 7.97E-10 |  | *RNF20* | 0.251 | 2.90E-07 |
| *NRIP1* | 0.299 | 8.07E-10 |  | *NEXN* | 0.251 | 2.90E-07 |
| *DEK* | 0.299 | 8.10E-10 |  | *RRP15* | 0.251 | 2.94E-07 |
| *RNF19B* | 0.299 | 8.33E-10 |  | *TLR8* | 0.251 | 2.98E-07 |
| *BEST1* | 0.299 | 8.38E-10 |  | *TAF6* | 0.251 | 3.00E-07 |
| *PCMT1* | 0.299 | 8.43E-10 |  | *LYAR* | 0.251 | 3.02E-07 |
| *SDS* | 0.298 | 8.46E-10 |  | *CHN1* | 0.251 | 3.03E-07 |
| *FGF7* | 0.298 | 8.57E-10 |  | *PI15* | 0.251 | 3.06E-07 |
| *TEAD4* | 0.298 | 8.71E-10 |  | *BLZF1* | 0.251 | 3.07E-07 |
| *HNRNPR* | 0.298 | 8.78E-10 |  | *DIAPH1* | 0.251 | 3.10E-07 |
| *NDC1* | 0.298 | 8.83E-10 |  | *AP4E1* | 0.251 | 3.14E-07 |
| *MCUB* | 0.298 | 8.88E-10 |  | *RANBP2* | 0.251 | 3.15E-07 |
| *THBS2* | 0.298 | 8.89E-10 |  | *KIF2C* | 0.250 | 3.19E-07 |
| *NOCT* | 0.298 | 8.99E-10 |  | *COL10A1* | 0.250 | 3.20E-07 |
| *WAC* | 0.298 | 9.16E-10 |  | *IGDCC4* | 0.250 | 3.22E-07 |
| *ACLY* | 0.298 | 9.17E-10 |  | *USP25* | 0.250 | 3.23E-07 |
| *CENPA* | 0.298 | 9.21E-10 |  | *KIFAP3* | 0.250 | 3.25E-07 |
| *TMEM200A* | 0.298 | 9.31E-10 |  | *PNPT1* | 0.250 | 3.28E-07 |
| *GOLGA7* | 0.298 | 9.33E-10 |  | *CTH* | 0.250 | 3.29E-07 |
| *APP* | 0.298 | 9.46E-10 |  | *POLR1A* | 0.250 | 3.32E-07 |
| *FAM220A* | 0.298 | 9.48E-10 |  | *TNFAIP8L3* | 0.250 | 3.32E-07 |

| **Supplementary Table 2. The list of top 10 immunologic signature gene sets positively associated with HBP** | | | | | | |  |
| --- | --- | --- | --- | --- | --- | --- | --- |
| **GS follow link to MSigDB** | **Size** | **ES** | **NES** | **Nominal p-value** | **FDR** | **Rank at Max** | |
| PATEL_SKIN_OF_BODY_ZOSTAVAX_AGE_70  _93YO_VZV_CHALLENGE_6HR_UP | 288 | 0.628 | 2.277 | 0 | 0 | 3526 | |
| GSE30971_CTRL_VS_LPS_STIM_MACROPHAGE  _WBP7_HET_2H_UP | 184 | 0.588 | 2.256 | 0 | 8.66E-04 | 4030 | |
| GSE1460_NAIVE_CD4_TCELL_ADULT_BLOOD _VS_THYMIC_STROMAL_CELL_DN | 197 | 0.511 | 2.212 | 0 | 0.00416 | 2190 | |
| GSE39556_CD8A_DC_VS_NK_CELL_UP | 199 | 0.515 | 2.208 | 0 | 0.00339 | 3492 | |
| GSE1460_INTRATHYMIC_T_PROGENITOR  _VS_THYMIC_STROMAL_CELL_DN | 196 | 0.547 | 2.1457 | 0 | 0.01831 | 2942 | |
| GSE9988_ANTI_TREM1_  VS_VEHICLE_TREATED_MONOCYTES_UP | 183 | 0.541 | 2.136 | 0 | 0.01834 | 4210 | |
| GSE30971_CTRL_VS  _LPS_STIM_MACROPHAGE_WBP7_KO_4H_UP | 188 | 0.539 | 2.109 | 0 | 0.02553 | 4240 | |
| GSE45739_NRAS_KO_VS  _WT_ACD3_ACD28_STIM_CD4_TCELL_UP | 194 | 0.547 | 2.066 | 0 | 0.04761 | 3543 | |
| GSE1460_CD4_THYMOCYTE_VS  _THYMIC_STROMAL_CELL_DN | 197 | 0.51 | 2.066 | 0 | 0.04232 | 3531 | |
| GSE23114_WT_VS  _SLE2C1_MOUSE_SPLEEN_B1A_BCELL_UP | 197 | 0.542 | 2.065 | 0 | 0.03897 | 4366 | |
